# The Regulatory Network and Potential Role of LINC00973-miRNA-mRNA ceRNA in the Progression of Non-Small-Cell Lung Cancer

**DOI:** 10.3389/fimmu.2021.684807

**Published:** 2021-07-29

**Authors:** Qiang Guo, Dan Li, Xiangyu Luo, Ye Yuan, Tian Li, Huasong Liu, Xinju Wang

**Affiliations:** ^1^Department of Cardiothoracic Surgery, Taihe Hospital, Hubei University of Medicine, Shiyan, China; ^2^Department of Oncology, Huanggang Central Hospital, Huanggang, China; ^3^School of Basic Medicine, Fourth Military Medical University, Xi’an, China; ^4^Department of Respiratory, Xinchang People’s Hospital, Xinchang, China

**Keywords:** LINC00973, non-small cell lung cancer, prognosis, biomarker, ceRNA

## Abstract

**Background:**

The occurrence and development of cancer could be promoted by abnormally competing endogenous RNAs (ceRNA) network. This article aims to determine the prognostic biomarker of ceRNA for non-small-cell lung cancer (NSCLC) prognosis.

**Methods:**

The expression and clinical significance of LINC00973 in NSCLC tissues were analyzed *via* the The Cancer Genome Atlas (TCGA), Gene Expression Profiling Interactive Analysis (GEPIA), lnCAR, and clinical samples in Taihe Hospital. The biological functions and signaling pathways involved in target genes of ceRNA network were analyzed *via* Gene Ontology (GO) and Kyoto Encyclopedia of Genes and Genomes (KEGG). Survival analysis, univariate and multivariate Cox regression analysis were used for prognostic-related mRNA.

**Results:**

Expression of LINC00973 was increased in NSCLC tissues. High expression of LINC00973 was associated with poor prognosis of NSCLC patients. There were 15 miRNA and 238 differential mRNA in the INC00973-miRNA-mRNA ceRNA network, involving cell migration, endothelial cell proliferation, tumor growth factor (TGF)-β, cellular senescence, phosphatidylinositol 3-hydroxy kinase (PI3K)-Akt, Hippo, Rap1, mitogen-activated protein kinase (MAPK), cell cycle signaling pathway, etc. The expression levels of RTKN2, NFIX, PTX3, BMP2 and LOXL2 were independent risk factors for the poor prognosis of NSCLC patients.

**Conclusions:**

LINC00973-miRNA-mRNA ceRNA network might be the basis for determining pivotal post-translational regulatory mechanisms in the progression of NSCLC. BMP2, LOXL2, NFIX, PTX3 and RTKN2 might be valuable prognostic markers and potential therapeutic targets.

## Introduction

According to Global Cancer Statistics 2020, with an estimated 2.2 million new cancer cases and 1.8 million deaths, lung cancer was the second most commonly diagnosed cancer and the leading cause of cancer death in 2020, representing approximately one in 10 (11.4%) cancers diagnosed and one in 5 (18.0%) deaths (1–4). Lung cancer included non-small cell carcinoma (NSCLC) and small cell carcinoma. At present, surgery was the first choice for NSCLC patients at early-stage, and combined therapy was the main way for NSCLC patients at middle and advanced stage (5). With the improvement of treatment methods, the prognosis of cancer patients was improved, but the overall survival (OS) of NSCLC patients remained still frustrating. Recently, the researches had provided new insights into the molecular mechanisms of NSCLC in genomics and transcriptomics. For example, LCAT1, a member of long non-coding RNA (lncRNA), was an upregulated marker in lung cancer tissues. Elevated LCAT1 expression level was closely associated with poor prognosis of cancer patients (6). LncRNA MNX1-AS1 was upregulated in lung cancer tissues, and the prognosis of lung cancer patients with overexpression of MNX1-AS1 was often terrible. MNX1-AS1 promoted cell proliferation, migration and invasion of lung cancer. The MNX1-AS1/miR-527/BRF2 signaling axis was involved in the occurrence and development of lung cancer (7). The expression of LncRNA AFAP1-AS1 was increased in NSCLC tissues, and related to the TNM stage and tumor size of NSCLC patients. Interfering with the expression of AFAP1-AS1 could inhibit the growth of NSCLC cells *in vitro* and *in vivo* (8). Therefore, lncRNA played an essential role in the progression of lung cancer.

Studies have confirmed that non-coding RNA (ncRNA) played important roles in tumorigenesis and metastasis (6–8). microRNA (miRNA) and lncRNA belong to ncRNA, and both of which have miRNA recognition elements (MRE) (9, 10). miRNA could trigger degradation of downstream target genes by recognizing the MRE in the 3’non-transcribed region (11, 12). Therefore, when lncRNA and mRNA had the same MRE, both of them could competitively bind to miRNA, forming a competitive endogenous RNA (ceRNA) regulatory network (13, 14). When miRNA bound to the target mRNA, the stability of mRNA decreased and its translation was hindered, thereby affecting gene expression. When lncRNA bound to miRNA, the stability of competitive mRNA increases instead, so that transcription and translation were well performed. This was a competitive two-way gene expression regulation mechanism composed of endogenous RNA (15, 16).

The ceRNA network mechanism presents a significant role in the occurrence and development of cancer. For example, the expression of circ_0025033 and small nuclear ribonucleoprotein Sm-like4 (LSM4) increased in ovarian cancer tissues and cells. Interfered with circ_0025033 or LSM4 expression could inhibit colony formation, migration, invasion and glycolytic metabolism of ovarian cancer cells. Circ_0025033 acted as a ceRNA to regulate LSM4 expression by targeting miR-184. Overexpression of LSM4 promoted the expression of circ_0025033, thereby inducing colony formation, migration, invasion and glycolysis (17). This indicated that there were abnormalities in the ceRNA network mechanism in the development of cancer. It was recently discovered that lncRNA LINC00973 had a vital effect in cancer. For example, chemotherapy could upregulate the expression level of LINC00973 in normal cells and cancer cells, which might be related to the activation of DNA damage response pathways or mitotic arrest. LINC00973 could reduce p21 levels, activate cancer cell proliferation, and reduce the lethality of drugs (18). Siglec-15 was a tumor immunosuppressive molecule. LINC00973 was highly expressed in Siglec-15 positive clear cell renal cell carcinoma, and it could positively regulate the expression of Siglec-15, which was the direct target molecule of miR-7109. LINC00973 could regulate the expression of Siglec-15 by affecting the role of sponge miR-7109 and forming ceRNA. Therefore, the LINC00973-miR-7109-Siglec-15 ceRNA mechanism was involved in regulating the progression of renal cell carcinoma (19). Nowadays, there was no related literature report about the regulation mechanism of ceRNA network composed of LINC00973 in the progress of NSCLC. Therefore, this study aims to explore the role and potential value of LINC00973-miRNA-mRNA ceRNA in the progression of NSCLC, and to provide new target molecules and potential regulatory mechanisms for NSCLC diagnosis and treatment.

## Materials and Methods

### NSCLC Tissue Sample

Cancer and normal adjacent tissues of 25 patients with NSCLC undergoing surgical treatment were collected at the Department of Thoracic Surgery, Taihe Hospital from December 2019 to April 2020. Inclusion criteria: (1) NSCLC was diagnosed pathologically; (2) Neoadjuvant chemotherapy or radiotherapy was not performed before surgery; (3) Immunosuppressant therapy, biological therapy, or targeted therapy were not performed. Exclusion criteria: 1) SCLC; 2) Patients voluntarily withdraw. This study was approved by the ethics committee of the Taihe Hospital, Hubei University of Medicine, and the patient has signed an informed consent. Among them, 11 cases were lung adenocarcinoma (LUAD) and 14 cases were lung squamous cell carcinoma (LUSC).

### TCGA Database

In October 2020, 1145 samples were downloaded from the TCGA (https://portal.gdc.cancer.gov/) website (108 cases of normal lung tissue, including 59 cases of normal lung tissue from LUAD and 49 cases of normal lung tissue from LUSC; 1037 cases of NSCLC tissue samples, including 535 samples of LUAD Cases and 522 cases of LUSC samples) HTSeq-FPKM transcriptome data and TCGA clinical data of 1026 NSCLC patients. The clinical parameters and prognostic information of NSCLC patients was sorted out. Patients with unknown or incomplete parameters to analyze the relationship between the expression level of LINC00973 and the prognosis was excluded. The limma package was used to analyze the differential expression genes in the tissues of NSCLC patients, and the screening conditions: fold change > 1 and P < 0.05.

### Quantitative Real-Time PCR

According to the RNA kit (Invitrogen, USA) instructions, total RNA was extracted from NSCLC tissue, and the qualified RNA concentration was detected. Copying DNA (cDNA) was synthesized using a reverse transcription kit (Takara, Japan), and the expression of LINC00973 was detected by quantitative real-time PCR (qRT-PCR). Primers was supplied by Sangong Co Ltd. GAPDH was used as an internal control. Primer sequence (5’-3’): LINC00973: TTGAAGGCTTCCTGGTCTGAG (Forward), AGGCTTACATTCCAGCTGTGT (Reverse), GAPDH: AACGGATTTGGTCGTATTG (Forward), GGAAGATGGTGATGGGATT (Reverse). Experiments were performed in triplicate independently.

### LNCAR and GEPIA Database

The LNCAR (http://lncar.renlab.org/) database data were acquired from the GEO database, which contained the cancer-related lncRNA expression database. The expression of LINC00973 in lung cancer tissues and its potential clinical value were analyzed in the LNCAR database. The screening criteria: P < 0.05. The GEPIA (http://gepia.cancer-pku.cn/) database was a reanalysis online database based on the TCGA and GTEx database data. The expression of LINC00973 and biomarkers in ceRNA network were explored. And the relationship between expression of LINC00973 and NSCLC overall survival (OS) and disease-free survival (DFS) were analyzed in the GEPIA database.

### ceRNA Network

The miRNA of LINC00973 were predicted in the LncBase Predicted v.2 database, and the screening criteria: binding coefficient ≥ 0.6. In addition, the miRNA that predicting the differentially expressed genes of TCGA database were performed *via* the PITA, RNA22, miRmap, microT, miRanda, PicTar, and TargetScan databases, and the screening criteria: the number of databases with targeting relationships needed to be greater than 3. Screening for overlapping miRNA in the LINC00973 target miRNA and miRNA that differentially expressed genes of TCGA database, and the ceRNA network signaling mechanism was constructed *via* Cytoscape 3.6.1 software.

### Gene Ontology (GO) and Kyoto Encyclopedia of Genes and Genomes (KEGG)

LncRNA competitively inhibited the mRNA binding to miRNA, and could maintain the stability of mRNA and promotes RNA transcription when LncRNA binds to miRNA. Therefore, the biological functions and signaling mechanisms involved in the ceRNA network was explored *via* the R clusterProfiler package. GO annotation included the biological process (BP), molecular function (MF) and cellular component (CC). The screening criteria: P < 0.05.

### Prognostic Survival Analysis

Kaplan-Meier survival analysis was used to evaluate the role of differentially expressed genes in the prognosis of NSCLC patients to understand the important target molecules in the ceRNA mechanism. The screening criteria was P < 0.05. On this basis, univariate Cox and multivariate Cox regression analysis were performed, and a nomogram was constructed.

### The Value of Poor Prognostic Factors in NSCLC Patients Were Verified

The GEPIA database was used to verify the expression of poor prognostic factors in the ceRNA network in NSCLC tissues. In addition, the Cbioportal database (http://www.cbioportal.org) was used to explore the relationship between poor prognostic factors and clinical pathological characteristics of patients with NSCLC. PrognoScan (http://www.abren.net/PrognoScan/) and Kaplan-Meier Plotter (http://kmplot.com/analysis/) databases were used to verify the value of poor prognostic factors in the prognosis of NSCLC patients, and the grouping standard was according to the best cutoff value.

### Statistical Analysis

Data processing and statistical analysis adopt perl and R language. The expression level and prognostic value of LINC00973 and mRNA in the ceRNA network were analyzed by the wilcoxon signed-rank test and Kaplan-Meier survival analysis. In addition, the role of differentially expressed genes in NSCLC prognosis in the ceRNA network were explored *via* the univariate and multivariate Cox regression analysis. P < 0.05 was considered statistically significant.

## Results

### LINC00973 Was Upregulated in NSCLC Tissues

**Figure 1:**
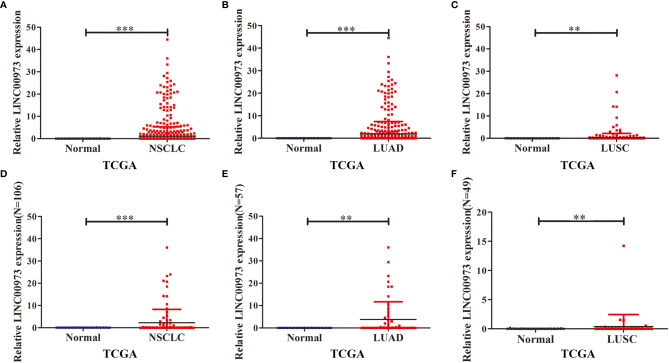
LINC00973 expression was elevated in NSCLC tissue in the TCGA database. **(A–C)** Expression of LINC00973 in unpaired samples of NSCLC, LUAD and LUSC; **(D–F)** Expression of LINC00973 in paired samples of NSCLC, LUAD and LUSC. NSCLC, Non-small cell lung cancer; LUAD, lung adenocarcinoma; LUSC, lung squamous cell carcinoma; Normal, normal tissues; TCGA, The Cancer Genome Atlas; **P < 0.01; ***P < 0.001.

In the TCGA database, the expression of LINC00973 was increased in unpaired NSCLC tissues (**Figure 1A**). The expression of LINC00973 was increased in unpaired LUAD and LUSC tissues (**Figures 1B, C**). The expression of LINC00973 in 106 paired NSCLC tissues was increased significantly (**Figure 1D**). The expression of LINC00973 was also increased in 57 paired LUAD and 49 paired LUSC significantly (**Figures 1E, F**).

**Figure 2:**
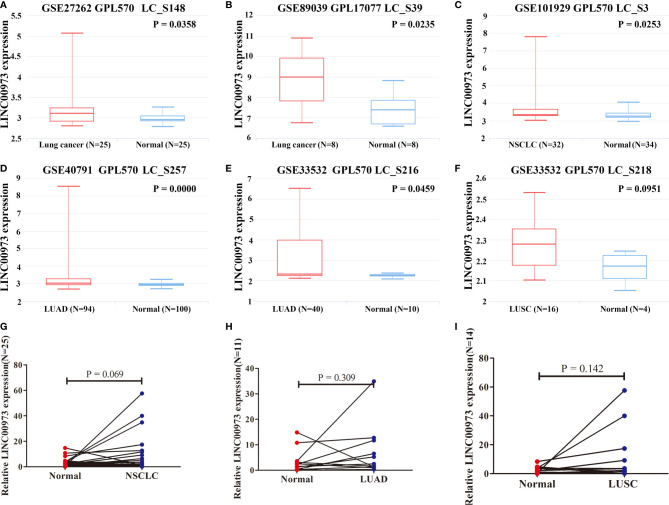
LINC00973 expression was upregulated in the LNCAR database and clinical NSCLC tissues. **(A)** GSE27262 (LC_S148); **(B)** GSE89039 (LC_S39); **(C)** GSE101929 (LC_S3); **(D)** GSE40791 (LC_S257); **(E)** GSE33532 (LC_S216); **(F)** GSE33532 (LC_S218); **(G)** Clinical NSCLC samples; **(H)** Clinical LUSD samples; **(I)** Clinical LUSCsamples. NSCLC, Non-small cell lung cancer; LUAD, lung adenocarcinoma; LUSC, lung squamous cell carcinoma; Normal, normal tissues.

In addition, the expression of LINC00973 was increased in NSCLC tissues in LNCAR database (**Figures 2A–F**). The GSE27262 and GSE89039 datasets showed that the expression of LINC00973 (LC_S148 and LC_S39) in lung cancer tissues was elevated, significantly (**Figures 2A, B**). The GSE101929 dataset revealed that the expression of LINC00973 (LC_S3) in NSCLC tissues was elevated (**Figure 2C**). The GSE40791 and GSE33532 datasets showed that the expression of LINC00973 (LC_S257 and LC_S216) in LUAD tissues was increased, and it was statistically significant (**Figures 2D, E**). The GSE33532 dataset showed that the expression of LINC00973 (LC_S218) in LUSC tissues was increased. However, it was not statistically significant (**Figure 2E**).

Moreover, we collected 25 clinical NSCLC tissues and adjacent normal lung tissues. We found that the expression of LINC00973 was increased in NSCLC and its subtypes *via* qRT-PCR. However, it was not statistically significant (**Figures 2G–I**).

### Elevated LINC00973 Expression Was Correlated With Poor Prognosis in NSCLC Patients

**Figure 3:**
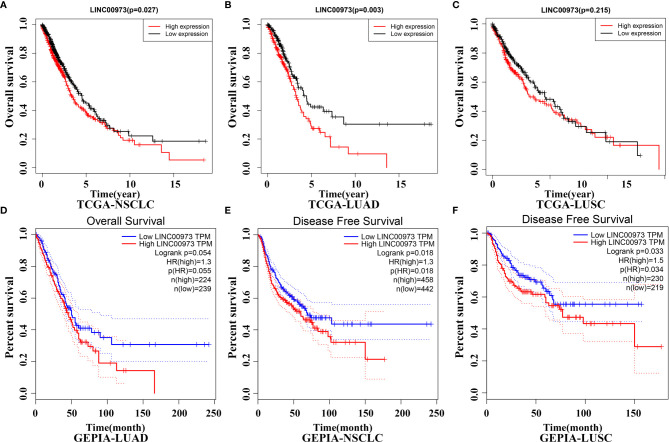
NSCLC patients with elevated lncRNA expression have poor prognosis in TCGA and GEPIA databases. **(A)** TCGA-NSCLC-OS; **(B)** TCGA-LUAD-OS; **(C)** TCGA-LUSC-OS; **(D)** GEPIA-LUAD-OS; **(E)** GEPIA-NSCLC-DFS; **(F)** GEPIA-LUSC-DFS. NSCLC, Non-small cell lung cancer; LUAD, lung adenocarcinoma; LUSC, lung squamous cell carcinoma; TCGA, The Cancer Genome Atlas; GEPIA, Gene Expression Profiling Interactive Analysis.

**Figure 4:**
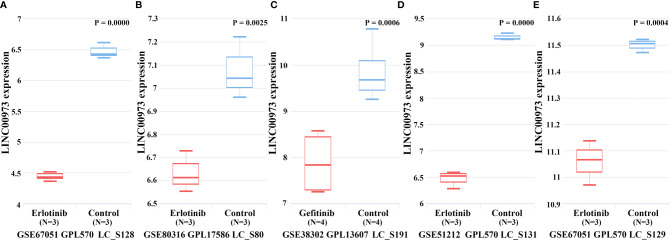
Anti-cancer drugs could down-regulate the expression of LINC00973. **(A)** GSE67051-erlotinib; **(B)** GSE80316-erlotinib; **(C)** GSE38302-gefitinib; **(D)** GSE51212-erlotinib; **(E)** GSE67051-erlotinib.

In the TCGA database, Kaplan-Meier survival curve suggested that increasing level of LINC00973 was closely associated with poor prognosis of NSCLC patients (**Figure 3A**). Subgroup analysis found that the prognosis of LUAD and LUSC patients with elevated LINC00973 expression levels were with poor prognosis, whereas there was no statistical significance between the LINC00973 expression levels and the prognosis of LUSC patients (**Figures 3B, C**). In addition, we found that increasing the expression levels of LINC00973 were with poor prognosis of NSCLC patients in the GEPIA database (**Figures 3D–F**). In detail, increasing levels of LINC00973 were negatively interrelated with OS and DFS in NSCLC patients, which indicated that LINC00973 might be a carcinogen. A consistent conclusion was reached in the LNCAR database. We found that anti-cancer drugs could inhibit the expression of LINC00973 in NSCLC PC9 and HCC827 cells (**Figure 4**). In detail, compared with the control group, GSE67051,GSE80316, and GSE51212 datasets showed that the expression of LINC00973 in the erlotinib treatment group was significantly decreased. The GSE38302 dataset showed that the expression of LINC00973 in the gefitinib group was significantly decreased.

### Construction of LINC00973-miRNA-mRNA ceRNA Network

**Figure 5:**
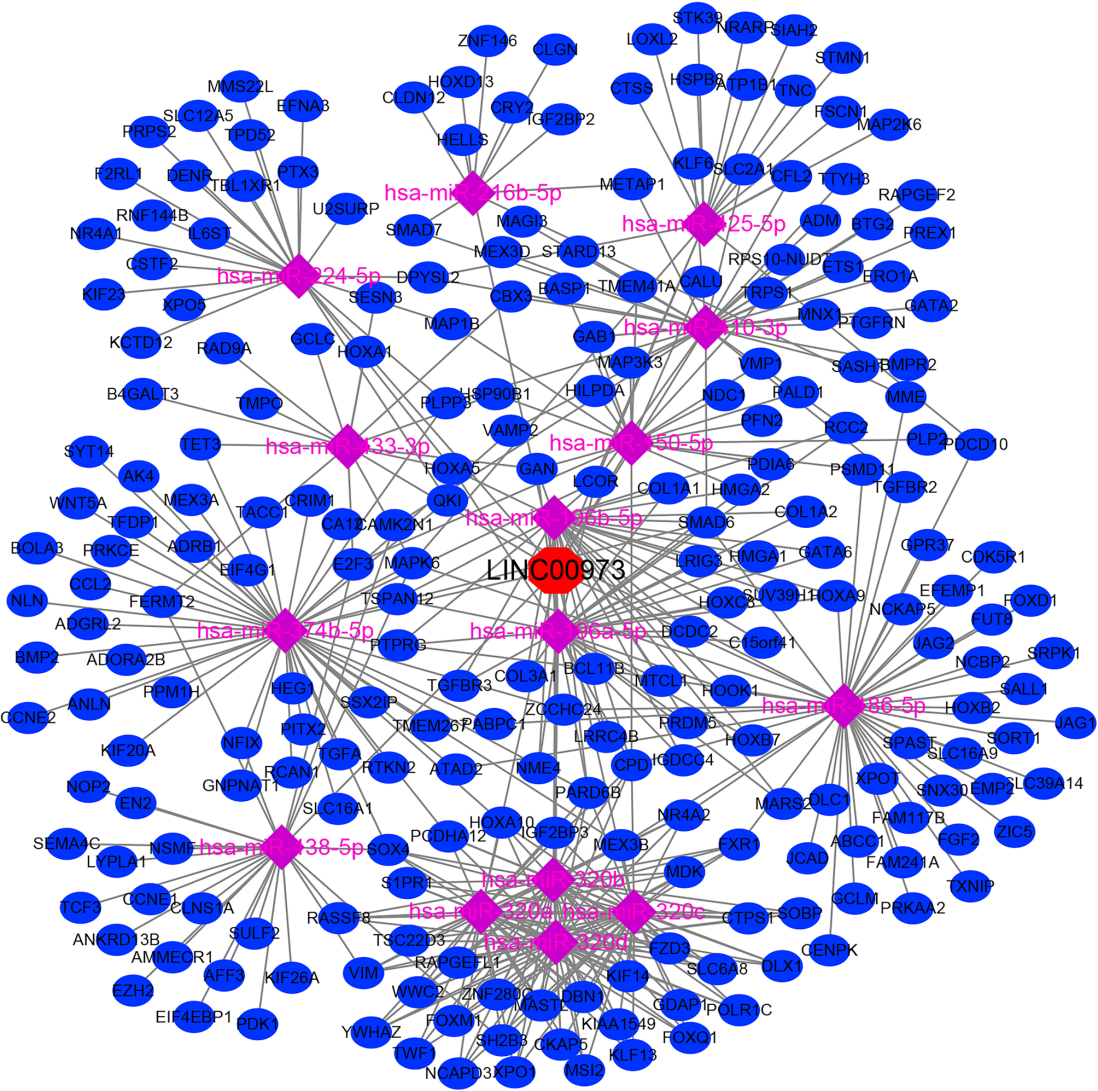
The LINC0097-miRNA-mRNA ceRNA network signaling mechanism.

In the LncBase Predicted v.2 database, a total of 210 miRNA were screened with the binding coefficient ≥ 0.6 (**Table 1**). In the TCGA database, 5367 differentially expressed genes were found in NSCLC tissues. We use heat map and violin map to show the top 20 differentially expressed genes in TCGA *via* fold changes (**Figures S1A, B**). We predicted that there were 232 miRNAs for differentially expressed genes (**Table S1**). Further comparison suggested that there were 15 miRNA that were competitively bound, namely hsa-miR-374b-5p, hsa-miR-216b-5p, hsa-miR-196a-5p, hsa-miR-320c, hsa-miR-138 -5p, hsa-miR-150-5p, hsa-miR-196b-5p, hsa-miR-224-5p, hsa-miR-320a, hsa-miR-186-5p, hsa-miR-320b, hsa-miR -425-5p, hsa-miR-410-3p, hsa-miR-320d and hsa-miR-433-3p (**Figure S2**). There were 238 target genes for miRNA in the ceRNA network (**Table S2**), and the LINC0097-miRNA-mRNA ceRNA network signaling mechanism was constructed (**Figure 5**).

**Table 1:** LINC00973-bound miRNA in the LncBase Predicted v.2 database.

LINC00973-miRNA				
hsa-miR-6749-3p	hsa-miR-5088-3p	hsa-miR-4700-5p	hsa-miR-513a-3p	hsa-miR-4633-5p
hsa-miR-3127-3p	hsa-miR-1238-3p	hsa-miR-133a-5p	hsa-miR-609	hsa-miR-374b-5p
hsa-miR-3177-5p	hsa-miR-3160-5p	hsa-miR-3180-5p	hsa-miR-433-3p	hsa-miR-6832-3p
hsa-miR-6875-3p	hsa-miR-541-5p	hsa-miR-4666a-5p	hsa-miR-7162-5p	hsa-miR-3151-3p
hsa-miR-6831-5p	hsa-miR-559	hsa-miR-33a-3p	hsa-miR-4719	hsa-miR-4694-3p
hsa-miR-6739-5p	hsa-miR-4778-3p	hsa-miR-6821-3p	hsa-miR-3121-3p	hsa-miR-216b-5p
hsa-miR-6756-3p	hsa-miR-6823-5p	hsa-miR-4282	hsa-miR-518d-3p	hsa-miR-208b-5p
hsa-miR-7113-5p	hsa-miR-629-3p	hsa-let-7f-1-3p	hsa-miR-579-3p	hsa-miR-4510
hsa-miR-545-3p	hsa-miR-5703	hsa-miR-4667-5p	hsa-miR-100-3p	hsa-miR-1238-5p
hsa-miR-449b-3p	hsa-miR-3153	hsa-miR-4501	hsa-miR-3689f	hsa-miR-4496
hsa-miR-4469	hsa-miR-520f-5p	hsa-miR-150-5p	hsa-miR-4474-3p	hsa-miR-138-5p
hsa-miR-6733-5p	hsa-miR-3973	hsa-miR-3668	hsa-miR-1915-5p	hsa-miR-6130
hsa-miR-320a	hsa-miR-3919	hsa-miR-3927-3p	hsa-miR-942-5p	hsa-miR-6892-3p
hsa-miR-320b	hsa-miR-6077	hsa-miR-188-3p	hsa-miR-3606-3p	hsa-miR-548as-5p
hsa-miR-4429	hsa-miR-6834-5p	hsa-miR-7853-5p	hsa-miR-7113-3p	hsa-miR-548d-5p
hsa-miR-532-3p	hsa-miR-6867-3p	hsa-miR-676-5p	hsa-miR-3678-3p	hsa-miR-5587-5p
hsa-miR-320e	hsa-miR-4786-3p	hsa-miR-2355-3p	hsa-miR-664b-3p	hsa-miR-6792-3p
hsa-miR-320d	hsa-miR-6839-5p	hsa-miR-376a-5p	hsa-miR-1207-3p	hsa-miR-1273g-5p
hsa-miR-320c	hsa-miR-369-3p	hsa-miR-1277-5p	hsa-miR-518b	hsa-miR-6127
hsa-miR-675-3p	hsa-miR-4677-3p	hsa-miR-4760-3p	hsa-miR-3617-3p	hsa-miR-7843-3p
hsa-miR-4679	hsa-miR-2276-5p	hsa-miR-4455	hsa-miR-3123	hsa-miR-548a-5p
hsa-miR-3680-3p	hsa-miR-4434	hsa-let-7a-3p	hsa-miR-548b-5p	hsa-miR-6129
hsa-miR-493-5p	hsa-miR-7156-3p	hsa-miR-105-5p	hsa-miR-7154-5p	hsa-miR-4691-5p
hsa-miR-6780b-5p	hsa-miR-6772-5p	hsa-miR-520h	hsa-miR-224-5p	hsa-miR-6875-5p
hsa-miR-4725-3p	hsa-miR-509-5p	hsa-miR-5701	hsa-miR-4762-3p	hsa-miR-6133
hsa-miR-5692c	hsa-miR-3925-5p	hsa-miR-539-5p	hsa-miR-148b-5p	hsa-miR-548k
hsa-miR-5692b	hsa-miR-297	hsa-miR-3662	hsa-miR-516b-3p	hsa-miR-548j-5p
hsa-miR-3922-5p	hsa-miR-509-3-5p	hsa-miR-4753-5p	hsa-miR-516a-3p	hsa-miR-3126-5p
hsa-miR-3671	hsa-miR-4287	hsa-miR-2054	hsa-miR-517-5p	hsa-miR-378j
hsa-miR-1252-3p	hsa-miR-3646	hsa-miR-3149	hsa-miR-518c-3p	hsa-miR-4419a
hsa-miR-568	hsa-miR-4650-3p	hsa-miR-3942-3p	hsa-miR-425-5p	hsa-miR-548av-5p
hsa-miR-2115-5p	hsa-miR-6512-3p	hsa-miR-520g-3p	hsa-miR-612	hsa-miR-6854-5p
hsa-miR-513b-3p	hsa-miR-6720-5p	hsa-miR-8061	hsa-miR-4662a-3p	hsa-miR-548i
hsa-miR-4731-5p	hsa-miR-4685-3p	hsa-miR-2116-3p	hsa-miR-4775	hsa-miR-548ao-5p
hsa-miR-8070	hsa-miR-4713-5p	hsa-miR-196b-5p	hsa-miR-4311	hsa-miR-186-5p
hsa-miR-4659b-3p	hsa-miR-410-3p	hsa-miR-580-5p	hsa-miR-4745-5p	hsa-miR-548ab
hsa-miR-4659a-3p	hsa-miR-7114-5p	hsa-miR-4320	hsa-miR-548p	hsa-miR-330-3p
hsa-miR-4666b	hsa-miR-205-3p	hsa-miR-148a-5p	hsa-miR-4516	hsa-miR-545-5p
hsa-miR-6758-3p	hsa-miR-4495	hsa-miR-4306	hsa-miR-3689e	hsa-miR-6815-3p
hsa-miR-670-3p	hsa-miR-4267	hsa-miR-513c-3p	hsa-miR-3689a-5p	hsa-miR-548c-5p
hsa-miR-5692a	hsa-miR-4271	hsa-miR-671-5p	hsa-miR-3689b-5p	hsa-miR-548o-5p
hsa-miR-4799-5p	hsa-miR-8089	hsa-miR-196a-5p	hsa-miR-4758-5p	hsa-miR-548am-5p

### The Biological Functions and Signaling Pathways Involved in the ceRNA Network Mechanism

**Figure 6:**
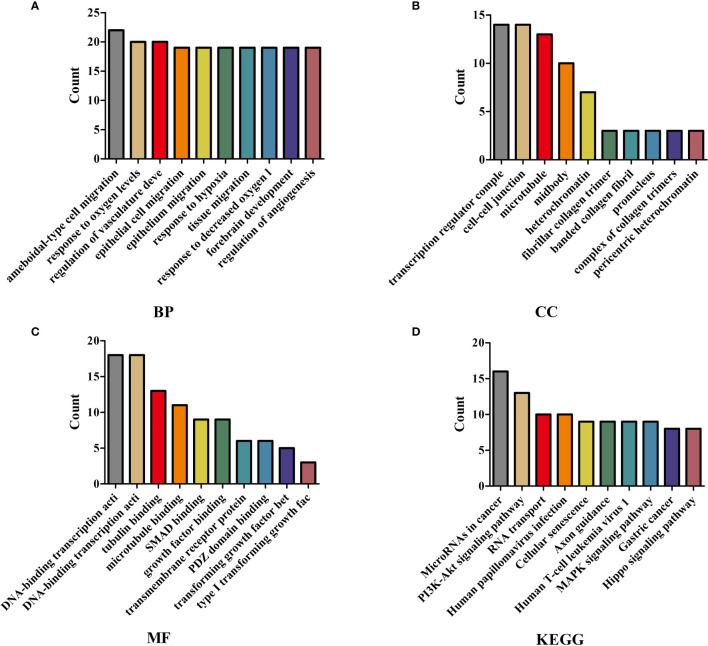
GO and KEGG showed that the biological functions and signaling pathways involved in the ceRNA network mechanism. **(A)** BP; **(B)** CC; **(C)** MF; **(D)** KEGG. GO, Gene Ontology; KEGG, Kyoto Encyclopedia of Genes and Genomes; BP, Biological process; CC, Cellular components; MF, Molecular function.

We performed GO annotation and KEGG on 238 mRNAs in the ceRNA network to explore the biological roles of LINC00973 of NSCLC. The GO annotation showed that LINC00973 might be involved in biological processes such as cell migration, response to oxygen levels, positive regulation of cell cycle process, and endothelial cell proliferation (**Figures 6A–C** and **Table S3**). KEGG showed that LINC00973 might be involved in the regulation of MicroRNAs in cancer, RNA transport, TGF β signaling pathway, Cellular senescence, PI3K-Akt signaling pathway, Hippo signaling pathway, Rap1 signaling pathway, Transcriptional misregulation in cancer, EGFR tyrosine kinase inhibitor resistance, MAPK signaling pathway, cell cycle and renin-angiotensin system and other signaling pathways (**Figure 6D** and **Table 2**).

**Table 2:** KEGG showed that the signaling pathways involved in the ceRNA network mechanism.

ID	Description	Count	P value
hsa05206	MicroRNAs in cancer	16	1.13E-05
hsa03013	RNA transport	10	0.000399544
hsa04350	TGF-beta signaling pathway	7	0.00044901
hsa04218	Cellular senescence	9	0.000470354
hsa04360	Axon guidance	9	0.001362992
hsa05226	Gastric cancer	8	0.001552477
hsa04151	PI3K-Akt signaling pathway	13	0.002058027
hsa04390	Hippo signaling pathway	8	0.002161407
hsa04933	AGE-RAGE signaling pathway in diabetic complications	6	0.003489698
hsa05166	Human T-cell leukemia virus 1 infection	9	0.004929187
hsa04550	Signaling pathways regulating pluripotency of stem cells	7	0.005076892
hsa04015	Rap1 signaling pathway	8	0.012196648
hsa05215	Prostate cancer	5	0.014040933
hsa00533	Glycosaminoglycan biosynthesis - keratan sulfate	2	0.017456577
hsa04974	Protein digestion and absorption	5	0.017798661
hsa05211	Renal cell carcinoma	4	0.018587208
hsa05224	Breast cancer	6	0.02124233
hsa04216	Ferroptosis	3	0.022153401
hsa05202	Transcriptional misregulation in cancer	7	0.023095703
hsa05165	Human papillomavirus infection	10	0.023906377
hsa04934	Cushing syndrome	6	0.0267241
hsa01521	EGFR tyrosine kinase inhibitor resistance	4	0.028904446
hsa04010	MAPK signaling pathway	9	0.029244879
hsa05161	Hepatitis B	6	0.032232807
hsa04110	Cell cycle	5	0.035980455
hsa05225	Hepatocellular carcinoma	6	0.03750644
hsa04614	Renin-angiotensin system	2	0.044544163

KEGG, Kyoto Encyclopedia of Genes and Genomes.

### Kaplan-Meier Survival Analysis Screens Prognostic Genes in the ceRNA Network

**Figure 7:**
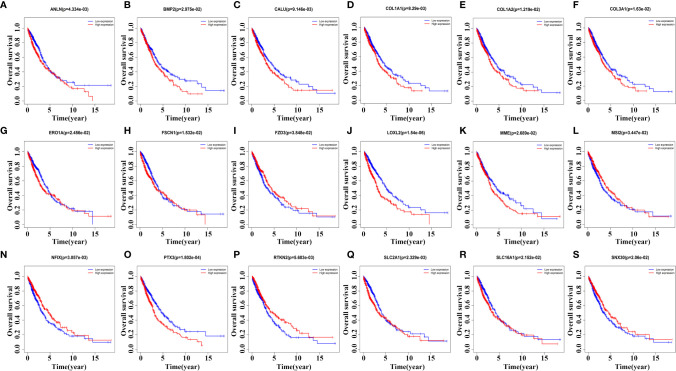
Kaplan-Meier survival analysis screens prognostic factors in the ceRNA network. **(A)** ANLN; **(B)** BMP2; **(C)** CALU; **(D)** COL1A1; **(E)** COL1A2; **(F)** COL3A1; **(G)** ERO1A; **(H)** FSCN1; **(I)** FZD3; **(J)** LOXL2; **(K)** MME; **(L)** MSI2; **(N)** NFIX; **(O)** PTX3; **(P)** RTKN2; **(Q)** SLC2A1; **(R)** SLC16A1; **(S)** SNX30.

Kaplan-Meier survival analysis revealed that the expression levels of ANLN, BMP2, CALU, COL1A1, COL1A2, COL3A1, ERO1A, FSCN1, FZD3, LOXL2, MME, MSI2, NFIX, PTX3, RTKN2, SLC2A1, SLC16A1, and SNX30 were relevant with poor prognosis in NSCLC patients (**Figure 7**). In detail, NSCLC patients with elevated expression levels of ANLN, BMP2, CALU, COL1A1, COL1A2, COL3A1, LOXL2, MME, PTX3 and SLC2A1 had a poor prognosis. The prognosis of NSCLC patients with reduced expression levels of FZD3, MSI2, NFIX, RTKN2 and SNX30 were poor, respectively (P < 0.05).

### Univariate and Multivariate Cox Regression Analysis to Screen Prognostic Factors in ceRNA Network

**Figure 8:**
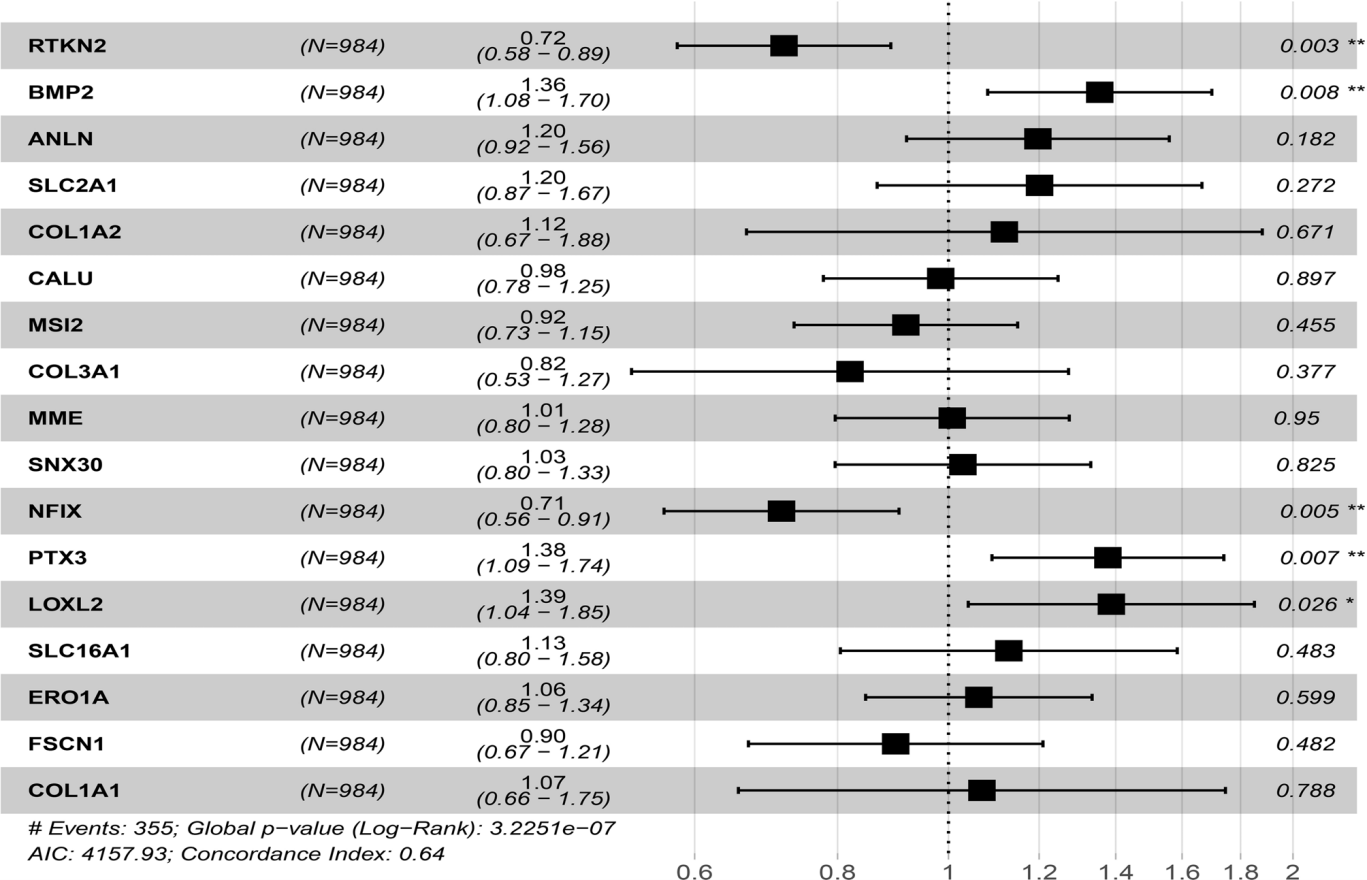
Univariate and multivariate cox regression analysis showed that prognostic factors of NSCLC patients in ceRNA network. *P < 0.05; **P < 0.01.

Univariate Cox regression analysis to screen 18 prognostic-related factors, it was found that LOXL2, PTX3, SLC2A1, RTKN2, ANLN, NFIX, CALU, ERO1A, BMP2, FSCN1, COL1A2, SLC16A1, COL3A1, MME, SNX30, COL1A1, and MSI2 might be prognostic risk factors of NSCLC patients (**Table 3**). Multivariate Cox regression analysis suggested that RTKN2, NFIX, PTX3, BMP2 and LOXL2 were independent risk factors for poor prognosis of NSCLC patients (**Figure 8**).

**Table 3:** Univariate Cox regression analysis showed that the prognostic factors of NSCLC patients in ceRNA. network.

Gene	HR	HR.95L	HR.95H	P value
RTKN2	0.742284429	0.60197367	0.915299456	0.005305366
BMP2	1.288159834	1.044214828	1.589094231	0.018085515
ANLN	1.344766746	1.088990666	1.660618091	0.005923755
SLC2A1	1.389067051	1.124226954	1.716296931	0.002327596
COL1A2	1.281970754	1.040016495	1.580214374	0.019931627
CALU	1.301912007	1.055942536	1.60517719	0.013531776
MSI2	0.808993441	0.656396957	0.997064931	0.046864172
COL3A1	1.262426589	1.023801232	1.556670224	0.029256551
MME	1.253713201	1.017413308	1.544895058	0.033839712
SNX30	0.797756898	0.646015773	0.985140141	0.035812917
NFIX	0.74784094	0.606606233	0.921958993	0.006511941
PTX3	1.490910274	1.208082379	1.839951881	0.000198204
LOXL2	1.660877052	1.344818347	2.051215756	2.47E-06
SLC16A1	1.279724346	1.036424381	1.580138822	0.021876855
ERO1A	1.295617838	1.05112084	1.596986301	0.015214925
FZD3	0.81542587	0.661816109	1.004688976	0.055360497
FSCN1	1.285122696	1.04084388	1.586732051	0.019693861
COL1A1	1.309300819	1.061681099	1.614673781	0.011750581

### Verified the Expression and Potential Clinical Value of Independent Risk Factors in NSCLC Tissues

**Figure 9:**
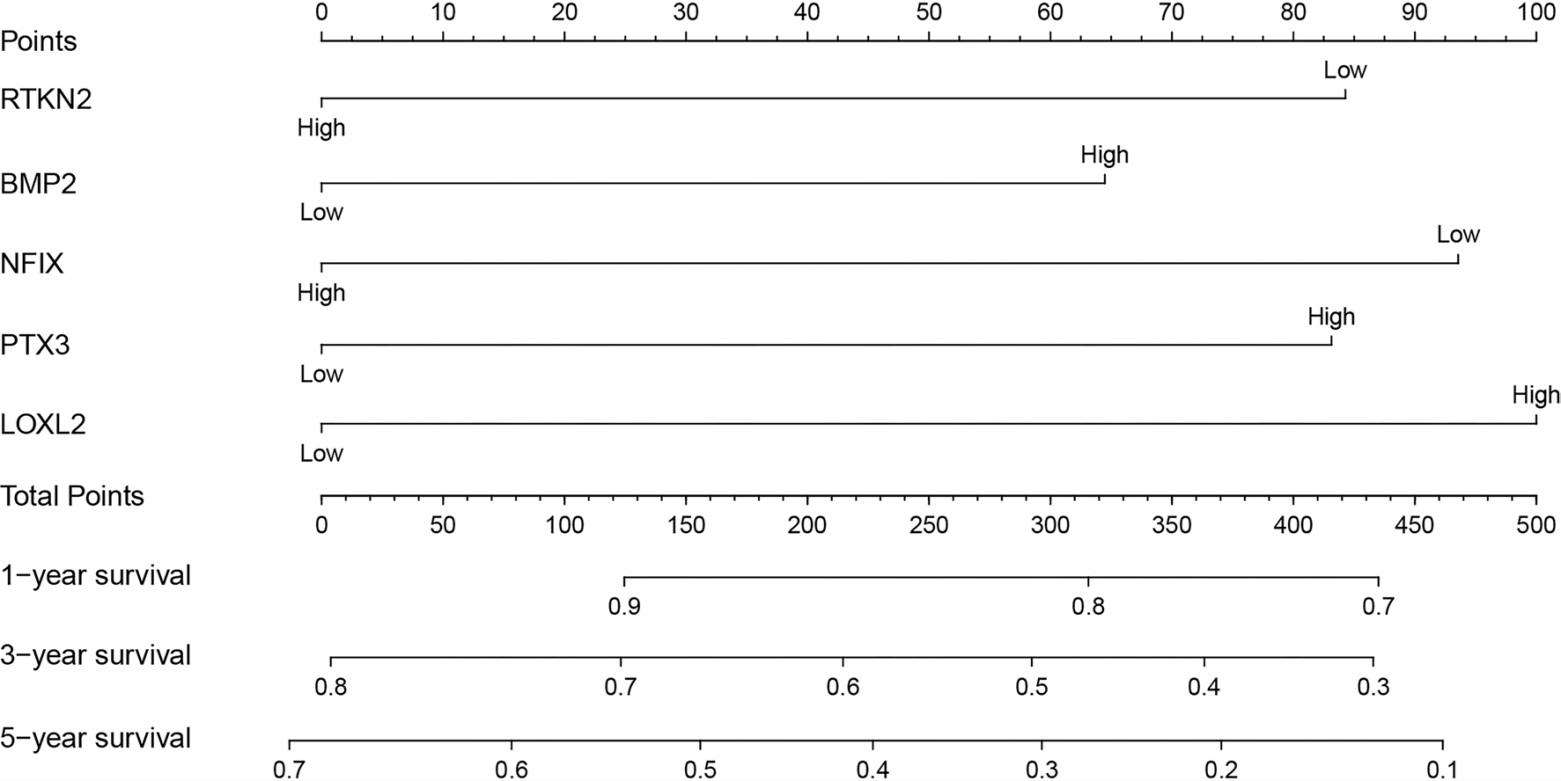
Alignment to construct prognostic related models.

In the GEPIA database, we found that the expression levels of RTKN2, NFIX, PTX3 and BMP2 in NSCLC subtypes LUAD and LUSC tissues were significantly declined, while the expression of LOXL2 in NSCLC tissues increased (**Figure S3**). **Figure S4** showed the relationship between RTKN2, NFIX, PTX3, BMP2, and LOXL2 mutation status and clinicopathological characteristics of NSCLC patients. In addition, the expression levels of RTKN2, NFIX, PTX3, BMP2 and LOXL2 were correlated with poor prognosis of NSCLC patients in the PrognoScan and Kaplan-Meier Plotter databases (**Figure S5** and **Table 4**). Therefore, we grouped the median values of RTKN2, NFIX, PTX3, BMP2, and LOXL2 expression to construct a nomogram to assess the prognosis of patients (**Figure 9**).

**Table 4:** The expression levels of RTKN2, NFIX, PTX3, BMP2 and LOXL2 were correlated with poor prognosis of NSCLC patients in the PrognoScan databases.

Gene	Dataset	Type	Endpoint	PROBE ID	N	COX P	HR [95% CI]
BMP2	GSE13213	LUAD	OS	A_23_P143331	117	2.69E-05	0.68 [0.57 - 0.81]
BMP2	GSE31210	LUAD	RFS	205290_s_at	204	0.0140761	0.67 [0.49 - 0.92]
BMP2	GSE31210	LUAD	RFS	205289_at	204	0.00186833	0.61 [0.45 - 0.83]
BMP2	GSE3141	NSCLC	OS	205289_at	111	0.0215927	1.34 [1.04 - 1.71]
NFIX	GSE13213	LUAD	OS	A_24_P858698	117	0.00181017	0.57 [0.40 - 0.81]
NFIX	GSE13213	LUAD	OS	A_23_P165295	117	0.0261232	0.71 [0.53 - 0.96]
NFIX	GSE31210	LUAD	RFS	209807_s_at	204	0.00254395	0.49 [0.31 - 0.78]
NFIX	GSE31210	LUAD	RFS	228278_at	204	0.000193836	0.51 [0.36 - 0.73]
NFIX	GSE31210	LUAD	OS	209807_s_at	204	0.00972759	0.45 [0.25 - 0.82]
NFIX	GSE31210	LUAD	OS	228278_at	204	0.0016013	0.48 [0.30 - 0.76]
NFIX	GSE8894	NSCLC	RFS	229834_at	138	0.040562	0.00 [0.00 - 0.59]
LOXL2	GSE31210	LUAD	OS	202998_s_at	204	0.0153133	1.78 [1.12 - 2.84]
LOXL2	GSE31210	LUAD	RFS	228808_s_at	204	0.044394	0.69 [0.48 - 0.99]
LOXL2	GSE31210	LUAD	RFS	202998_s_at	204	2.51E-05	2.12 [1.49 - 3.00]
LOXL2	GSE3141	NSCLC	OS	202998_s_at	111	0.00150164	1.95 [1.29 - 2.95]
LOXL2	GSE3141	NSCLC	OS	202997_s_at	111	0.0220928	1.41 [1.05 - 1.89]
LOXL2	GSE8894	NSCLC	RFS	202998_s_at	138	0.0103412	1.26 [1.06 - 1.49]
LOXL2	GSE8894	NSCLC	RFS	202997_s_at	138	0.000366017	2.21 [1.43 - 3.41]
PTX3	GSE31210	LUAD	OS	229760_at	204	0.0484883	0.76 [0.58 - 1.00]
PTX3	GSE31210	LUAD	RFS	229760_at	204	8.07E-05	0.67 [0.54 - 0.82]
PTX3	GSE31210	LUAD	RFS	229759_s_at	204	0.000904115	0.75 [0.63 - 0.89]
RTKN2	GSE31210	LUAD	RFS	230469_at	204	0.0162189	0.71 [0.54 - 0.94]
RTKN2	GSE3141	NSCLC	OS	230469_at	111	0.0421692	0.84 [0.70 - 0.99]

OS, Overall Survival; RFS, Relapse Free Survival; NSCLC, Non-small cell lung cancer; LUAD, lung adenocarcinoma; LUSC, lung squamous cell carcinoma.

## Discussion

Currently, it was urgent for us to improve the prognosis of NSCLC patients by finding novel and improved therapies. More and more evidences have showed that a large number of lncRNAs played extremely pivotal biological roles in the progression of NSCLC (20). For example, LncRNA KTN1-AS1, CASC9, ABHD11-AS1, LINC00467 could participate in the progress of NSCLC (20–23). Studies have reported that LINC00973 was related to cancer progression (18, 19, 24, 25). The expression level of LINC00973 was upregulated in cetuximab-resistant colorectal cancer H508/CR cells. Interfering the expression of LINC00973 decreased cell viability, increased cell apoptosis, and reduced glucose consumption and lactate secretion, which indicated that LINC00973 acted as a carcinogen (25). In addition, studies have reported that different doses of 5-fluorouracil, oxaliplatin, and irinotecan could impact the expression of LINC00973 in HT-29 and HCT-116 colon cancer cells. Combination medication increased the expression of LINC00973 in colon cancer cells (24). In our results, the expression of LINC00973 was elevated in NSCLC tissues. The high expression of LINC00973 was associated with poor prognosis in NSCLC patients, which indicated that LINC00973 might act as a carcinogen in the progression of NSCLC. As we all know, gefitinib and erlotinib were common anti-cancer drugs for lung cancer (26, 27), and Gefitinib and erlotinib could reduce the expression of carcinogenic LINC00973, which was expected to improve the prognosis of lung cancer patients. Therefore, LINC00973 might be a promising target for the development of new therapies for NSCLC.

lncRNAs could regulate the growth and migration behavior of cancer cells through a variety of mechanisms, including regulate expression of mRNA by ceRNA network, which could lead to its degradation (28, 29). For example, Cui et al. reported that the expression of lncRNA TRPM2-AS was significantly upregulated in NSCLC tissues and cells. Interfering with TRPM2-AS expression could inhibit cell proliferation, migration and invasion, and induce cell apoptosis. miR138-5p was the target downstream molecule of TRPM2-AS, and they showed a negative correlation. Interfering with TRPM2-AS expression could inhibit tumor formation, reduce the expression of EGFR and Ki67, and promote tumor cell apoptosis (28). At present, miR-374b-5p, miR-196a-5p, miR-138-5p, miR-150-5p, miR-196b-5p, miR-320a, miR-186-5p, etc. were related to the progress of NSCLC (30–38). For example, miR-138-5p could reduce the growth of NSCLC cells and increase the number of tumor-infiltrating dendritic cells (DCs). miR-138-5p down-regulates the expression of cyclin D3, CCD20, Ki67 and MCM in A549/3LL cells (37). The expression level of TSPAN12 was down-regulated in NSCLC tissues, and it was negatively correlated with the expression level of miR-196b-5p (35). However, LINC00973 was compatible with miR-374b-5p, miR-216b-5p, miR-196a-5p, miR-320c, miR-138-5p, miR-150-5p, miR-196b-5p, miR-224-5p. The relationship between miR-320a, miR-186-5p, miR-320b, miR-425-5p, miR-410-3p, miR-320d and miR-433-3p has not been reported yet, and we still need to conduct cell research to confirm.

RTKN2, NFIX, PTX3, BMP2 and LOXL2 of the ceRNA network play an important role in cancer progression (39–43). For instance, Ji et al. found that the expression level of RTKN2 in NSCLC tissues and cells was upregulated. Interfered with RTKN2 expression could induce NSCLC cell apoptosis and inhibit cell proliferation by increasing Bax levels and downregulating Bcl-2 levels. Interfered with RTKN2 expression could inhibit the migration and invasion of NSCLC cells by upregulating the expression of matrix metalloproteinase 9 (MMP9) and matrix metalloproteinase 2 (MMP2) (43). Huang et al. reported that elevated the levels of BMP2 were associated with short OS in NSCLC patients. BMP signaling was activated in the metastatic bone tumor of Lewis lung cancer in mice, and BMP2 signaling activation could enhance the bone metastasis of Lewis lung cancer, leading to poor prognosis. BMP2 secreted by mesenchymal fibroblasts could promote the migration and invasion of NSCLC cells (42). The expression of miR-504 in NSCLC tissues was significantly downregulated. The downregulation of miR-504 levels were positively correlated with lymph node metastasis and TNM stage. The overexpression of miR-504 significantly inhibited the proliferation, invasion and EMT process of NSCLC cells. miR-504 could bind to the 3’UTR region of LOXL2 and regulate its expression. Overexpression of LOXL2 could rescue the miR-504-induced inhibition of NSCLC cell proliferation and invasion (41). In our study, Univariate and multivariate Cox analysis found that the target molecules RTKN2, NFIX, PTX3, BMP2 and LOXL2 in the ceRNA network were independent risk factors for poor prognosis of NSCLC patients. This further showed that they had an important biological role in the progression of NSCLC.

In our study, we found that the expression of LINC00973 in NSCLC tissues was increased, and that the increased expression levels of LINC00973 were associated with the poor prognosis of NSCLC patients, and constructed the LINC00973-miRNA-mRNA ceRNA network competition mechanism. Most of the miRNA and mRNA in the competitive ceRNA network have been confirmed to have important biological roles in the progression of NSCLC. There were few studies on LINC00973 in cancer, but we first formally formalize the expression of LINC00973 in NSCLC tissues, and explore the ceRNA network regulation mechanism of LINC00973 to provide new candidate markers for the treatment of NSCLC. Then, our research needs to be verified by *in vivo* and *in vitro* experiments to confirm the role and value of LINC00973-miRNA-mRNA ceRNA in the progression of NSCLC.

## Conclusion

This study found that LINC00973-miRNA-mRNA ceRNA network might be the basis for determining important post-translational regulatory mechanisms in the progression of NSCLC. BMP2, LOXL2, NFIX, PTX3 and RTKN2 might be valuable prognostic markers and potential therapeutic targets in the progression of NSCLC.

## Data Availability Statement

The datasets presented in this study can be found in online repositories. The names of the repository/repositories and accession number(s) can be found in the article/**Supplementary Material**.

## Ethics Statement

The studies involving human participants were reviewed and approved by Ethics committee of the Affiliated Taihe Hospital of Hubei University of Medicine. The patients/participants provided their written informed consent to participate in this study.

## Author Contributions

HL, TL, and XW conceived the research topic, made the research plan and directed the implementation of the whole research. QG drafted the manuscript together and processed the data. DL assisted to collect and analyze the data. XL and YY performed data checking and language polishing. All authors contributed to the article and approved the submitted version.

## Conflict of Interest

The authors declare that the research was conducted in the absence of any commercial or financial relationships that could be construed as a potential conflict of interest.

## Publisher’s Note

All claims expressed in this article are solely those of the authors and do not necessarily represent those of their affiliated organizations, or those of the publisher, the editors and the reviewers. Any product that may be evaluated in this article, or claim that may be made by its manufacturer, is not guaranteed or endorsed by the publisher.

## Abbreviation

NSCLC, Non-small cell lung cancer; miRNA, microRNAs; LUAD, lung adenocarcinoma; LUSC, lung squamous cell carcinoma; lncRNA, long non-coding RNA; MRE, miRNA recognition elements; OS, overall survival.
