# Supplementary material for: The Regulatory Network and Potential Role of LINC00973-miRNA-mRNA ceRNA in the Progression of Non-Small-Cell Lung Cancer
Source: Front Immunol. 2021 Jul 29;12:684807. doi: 10.3389/fimmu.2021.684807 (PMC8358408; doi:10.3389/fimmu.2021.684807)
Supplement: Supplementary file 1 [file DataSheet_1.zip › Raw data of Fig 2/Figure 2A-E.docx]

Figure 2A-E data source was in the LNCAR online database (<http://lncar.renlab.org/>).

LINC00973 expression was upregulated in the LNCAR database. (A) GSE27262 (LC_S148); (B) GSE89039 (LC_S39); (C) GSE101929 (LC_S3); (D) GSE40791 (LC_S257); (E)GSE33532 (LC_S216); (F) GSE33532 (LC_S218).
