# Supplementary material for: The Regulatory Network and Potential Role of LINC00973-miRNA-mRNA ceRNA in the Progression of Non-Small-Cell Lung Cancer
Source: Front Immunol. 2021 Jul 29;12:684807. doi: 10.3389/fimmu.2021.684807 (PMC8358408; doi:10.3389/fimmu.2021.684807)
Supplement: Supplementary file 1 [file DataSheet_1.zip › Raw data of Fig 4/Figure 4.docx]

Figure 4 data source was in the LNCAR online database (<http://lncar.renlab.org/>).

Anti-cancer drugs could down-regulate the expression of LINC00973. (A) GSE67051-erlotinib; (B) GSE80316-erlotinib; (C) GSE38302-gefitinib; (D) GSE51212-erlotinib; (E) GSE67051-erlotinib.
