# Supplementary material for: The Regulatory Network and Potential Role of LINC00973-miRNA-mRNA ceRNA in the Progression of Non-Small-Cell Lung Cancer
Source: Front Immunol. 2021 Jul 29;12:684807. doi: 10.3389/fimmu.2021.684807 (PMC8358408; doi:10.3389/fimmu.2021.684807)
Supplement: Supplementary file 1 [file DataSheet_1.zip › Raw data of Fig S3/Figure S3.docx]

Figure S3 data source was in the GEPIA online database (http://gepia.cancer-pku.cn/).

Prognostic factors of NSCLC patients were differential expression. (A) TCGA; (B) TCGA+GTEx.
