# Supplementary material for: The Regulatory Network and Potential Role of LINC00973-miRNA-mRNA ceRNA in the Progression of Non-Small-Cell Lung Cancer
Source: Front Immunol. 2021 Jul 29;12:684807. doi: 10.3389/fimmu.2021.684807 (PMC8358408; doi:10.3389/fimmu.2021.684807)
Supplement: Supplementary file 1 [file DataSheet_1.zip › Raw data of Fig S5/Figure S5.docx]

Figure S5 data source was in the Kaplan-Meier Plotter online database (http://kmplot.com/analysis/).
