# Supplementary figures and images for: The Regulatory Network and Potential Role of LINC00973-miRNA-mRNA ceRNA in the Progression of Non-Small-Cell Lung Cancer
Source: Front Immunol. 2021 Jul 29;12:684807. doi: 10.3389/fimmu.2021.684807 (PMC8358408; doi:10.3389/fimmu.2021.684807)

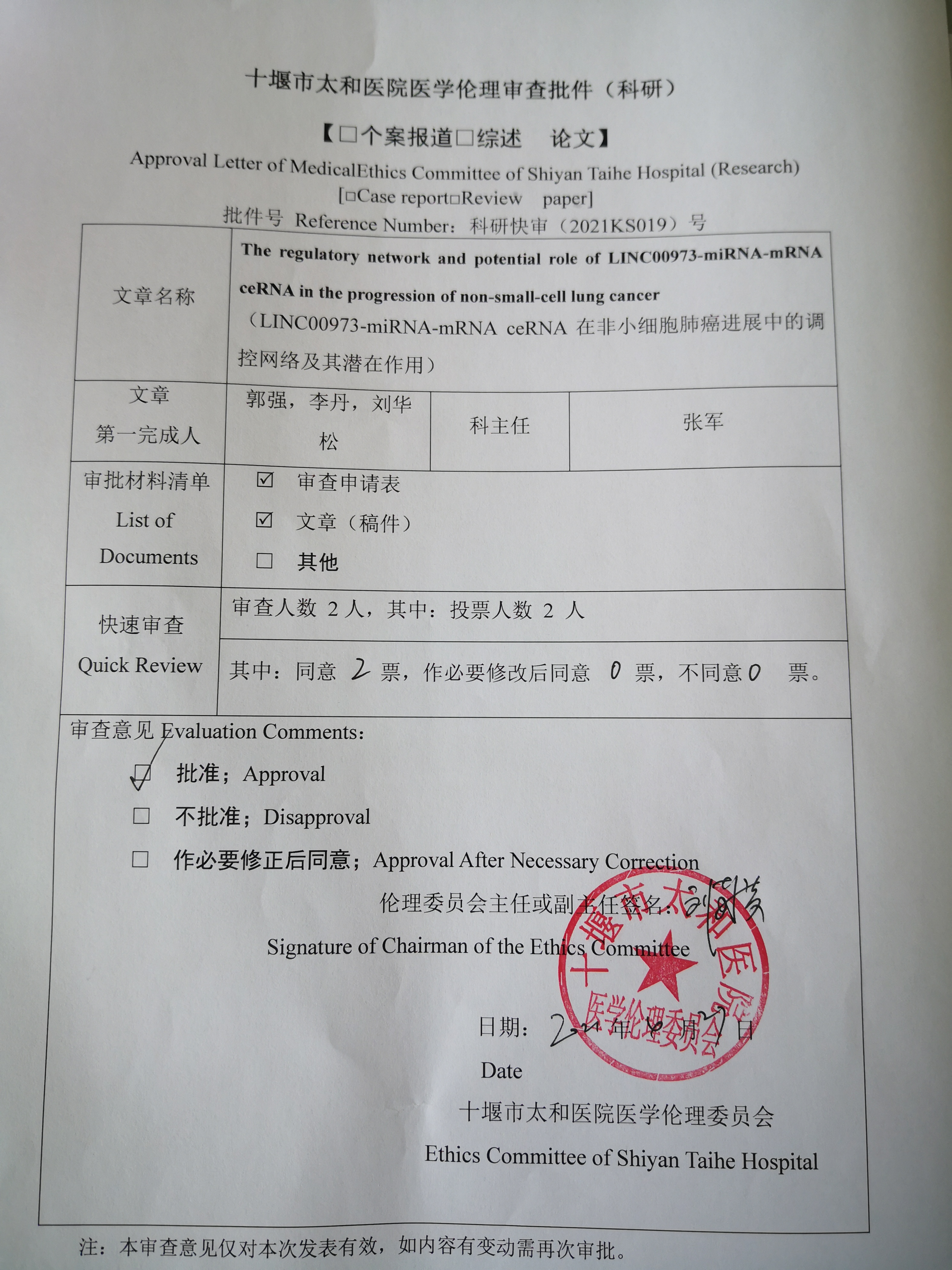

Supplement: Supplementary file 1 [file DataSheet_1.zip › Raw data of Fig 2/Ethical certification.jpg]

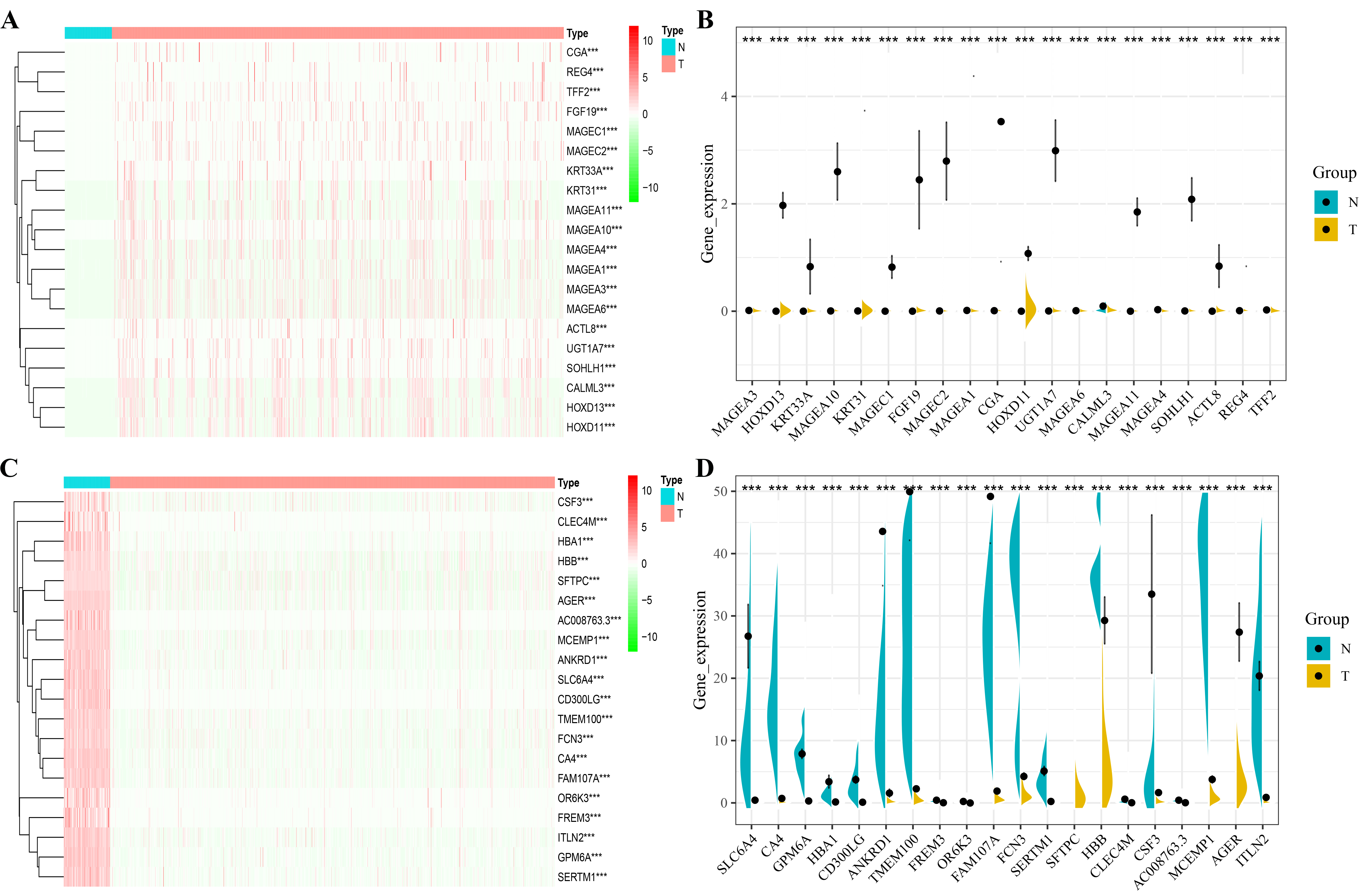

Supplement: Supplementary file 2 [file Image_1.jpg]

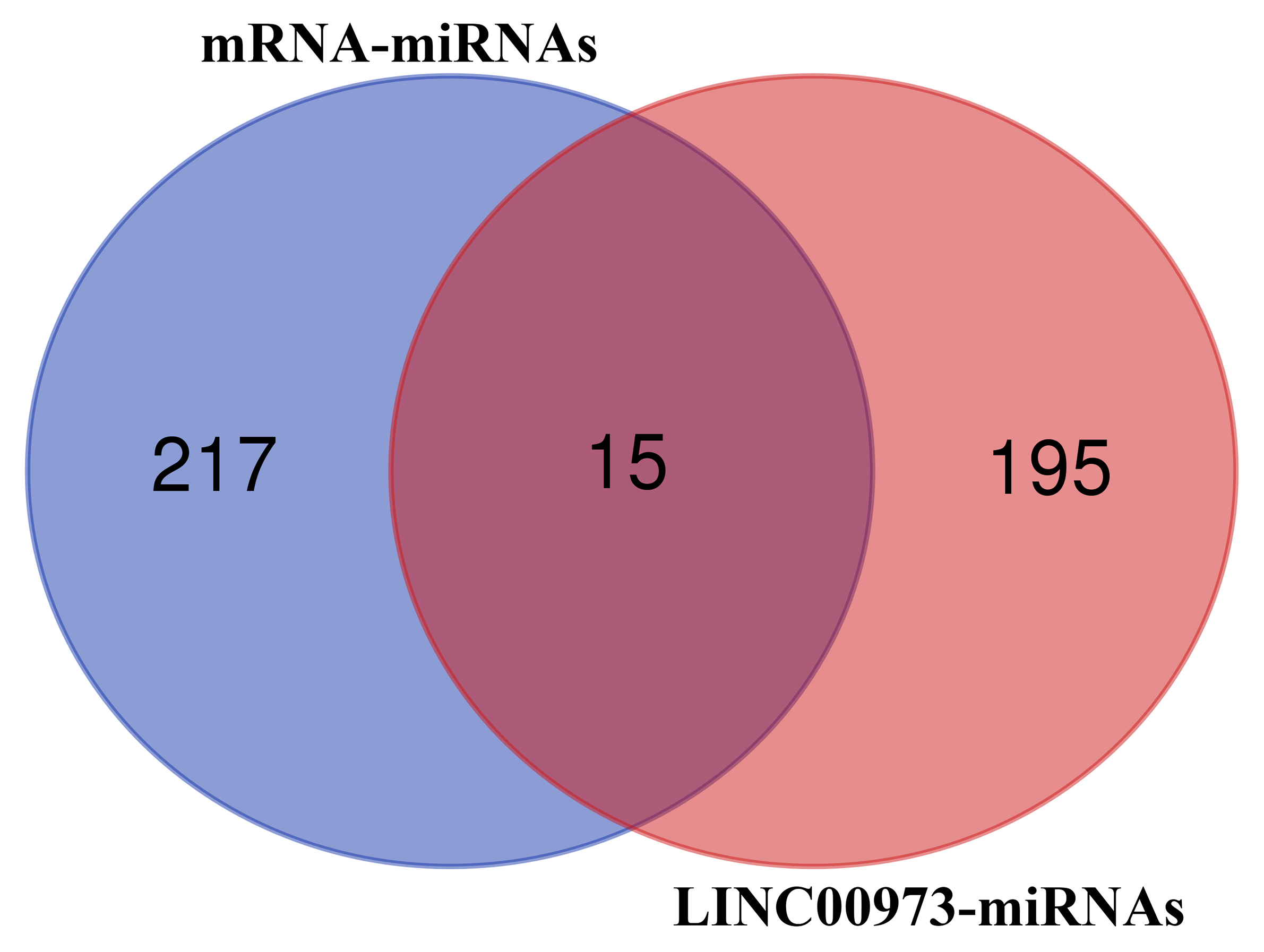

Supplement: Supplementary file 3 [file Image_2.jpg]

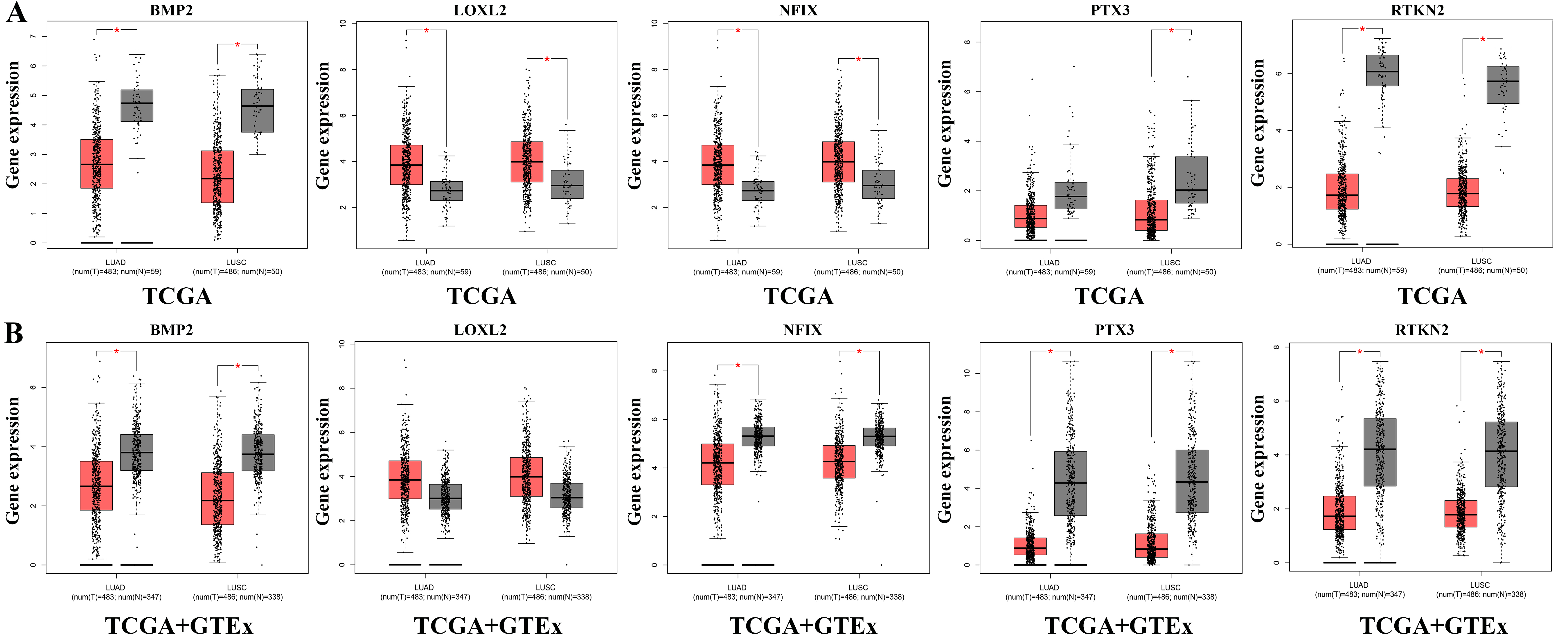

Supplement: Supplementary file 4 [file Image_3.jpg]

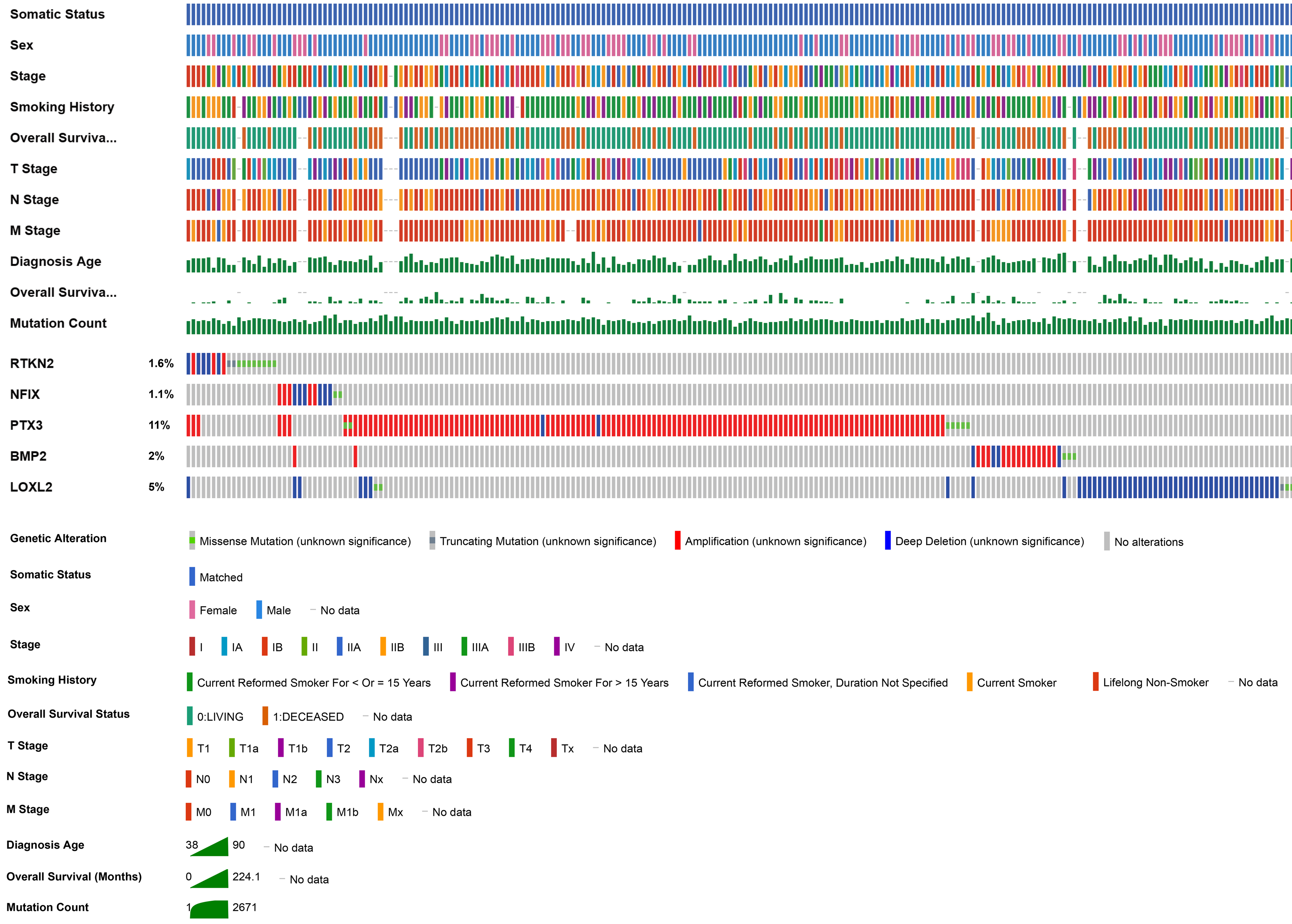

Supplement: Supplementary file 5 [file Image_4.jpg]

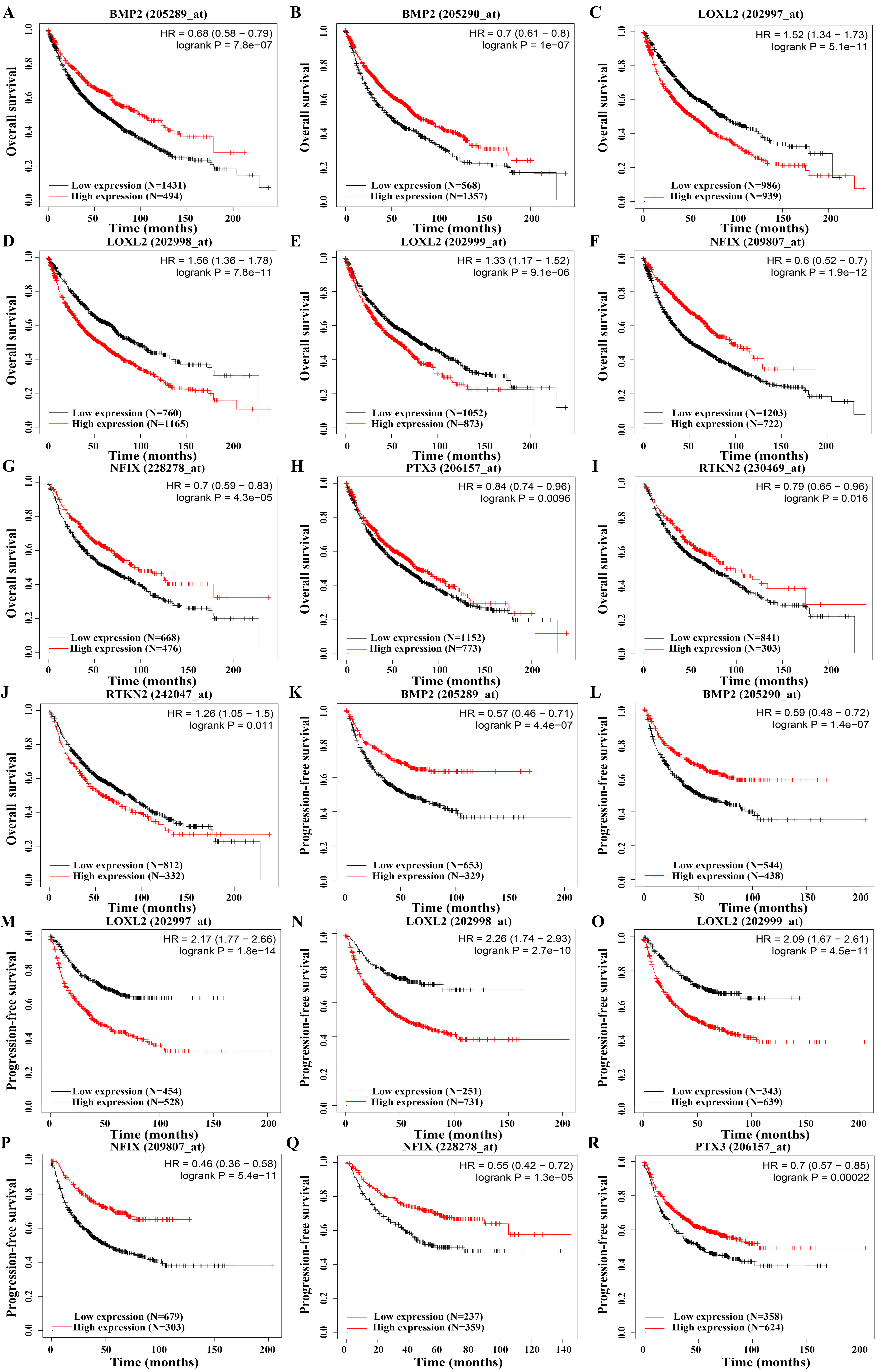

Supplement: Supplementary file 6 [file Image_5.jpg]
