# Supplementary material for: The Regulatory Network and Potential Role of LINC00973-miRNA-mRNA ceRNA in the Progression of Non-Small-Cell Lung Cancer
Source: Front Immunol. 2021 Jul 29;12:684807. doi: 10.3389/fimmu.2021.684807 (PMC8358408; doi:10.3389/fimmu.2021.684807)
Supplement: Supplementary file 7 [file Table_1.docx]

Supplementary Tables

Table S1. miRNA predicted by differentially expressed genes from the TCGA database.

| mRNA-miRNA | | | | | |
| --- | --- | --- | --- | --- | --- |
| hsa-let-7a-5p | hsa-miR-148b-3p | hsa-miR-210-3p | hsa-miR-320d | hsa-miR-433-3p | hsa-miR-140-5p |
| hsa-let-7b-5p | hsa-miR-149-5p | hsa-miR-211-5p | hsa-miR-324-5p | hsa-miR-448 | hsa-miR-141-3p |
| hsa-let-7c-5p | hsa-miR-150-5p | hsa-miR-212-3p | hsa-miR-326 | hsa-miR-449a | hsa-miR-142-3p |
| hsa-let-7d-5p | hsa-miR-152-3p | hsa-miR-215-5p | hsa-miR-328-3p | hsa-miR-449b-5p | hsa-miR-143-3p |
| hsa-let-7e-5p | hsa-miR-153-3p | hsa-miR-216a-5p | hsa-miR-329-3p | hsa-miR-451a | hsa-miR-144-3p |
| hsa-let-7f-5p | hsa-miR-154-5p | hsa-miR-216b-5p | hsa-miR-330-5p | hsa-miR-454-3p | hsa-miR-145-5p |
| hsa-let-7g-5p | hsa-miR-155-5p | hsa-miR-217 | hsa-miR-335-5p | hsa-miR-455-5p | hsa-miR-146a-5p |
| hsa-let-7i-5p | hsa-miR-15a-5p | hsa-miR-218-5p | hsa-miR-338-3p | hsa-miR-485-5p | hsa-miR-146b-5p |
| hsa-miR-1-3p | hsa-miR-15b-5p | hsa-miR-219a-5p | hsa-miR-339-5p | hsa-miR-486-5p | hsa-miR-148a-3p |
| hsa-miR-100-5p | hsa-miR-16-5p | hsa-miR-22-3p | hsa-miR-33a-5p | hsa-miR-488-3p | hsa-miR-200c-3p |
| hsa-miR-101-3p | hsa-miR-17-5p | hsa-miR-221-3p | hsa-miR-33b-5p | hsa-miR-490-3p | hsa-miR-204-5p |
| hsa-miR-103a-3p | hsa-miR-181a-5p | hsa-miR-222-3p | hsa-miR-340-5p | hsa-miR-491-5p | hsa-miR-205-5p |
| hsa-miR-106a-5p | hsa-miR-181b-5p | hsa-miR-223-3p | hsa-miR-342-3p | hsa-miR-494-3p | hsa-miR-206 |
| hsa-miR-106b-5p | hsa-miR-181c-5p | hsa-miR-224-5p | hsa-miR-346 | hsa-miR-495-3p | hsa-miR-208a-3p |
| hsa-miR-107 | hsa-miR-181d-5p | hsa-miR-23a-3p | hsa-miR-34a-5p | hsa-miR-496 | hsa-miR-208b-3p |
| hsa-miR-10a-5p | hsa-miR-182-5p | hsa-miR-23b-3p | hsa-miR-34c-5p | hsa-miR-497-5p | hsa-miR-20a-5p |
| hsa-miR-10b-5p | hsa-miR-183-5p | hsa-miR-24-3p | hsa-miR-361-5p | hsa-miR-499a-5p | hsa-miR-20b-5p |
| hsa-miR-122-5p | hsa-miR-184 | hsa-miR-25-3p | hsa-miR-362-3p | hsa-miR-503-5p | hsa-miR-21-5p |
| hsa-miR-124-3p | hsa-miR-185-5p | hsa-miR-26a-5p | hsa-miR-363-3p | hsa-miR-505-3p | hsa-miR-30c-5p |
| hsa-miR-125a-5p | hsa-miR-186-5p | hsa-miR-26b-5p | hsa-miR-365a-3p | hsa-miR-506-3p | hsa-miR-30d-5p |
| hsa-miR-125b-5p | hsa-miR-18a-5p | hsa-miR-27a-3p | hsa-miR-367-3p | hsa-miR-519d-3p | hsa-miR-30e-5p |
| hsa-miR-126-3p | hsa-miR-18b-5p | hsa-miR-27b-3p | hsa-miR-370-3p | hsa-miR-520a-3p | hsa-miR-31-5p |
| hsa-miR-1271-5p | hsa-miR-190a-5p | hsa-miR-28-5p | hsa-miR-371a-5p | hsa-miR-520b | hsa-miR-32-5p |
| hsa-miR-128-3p | hsa-miR-190b | hsa-miR-296-3p | hsa-miR-372-3p | hsa-miR-520c-3p | hsa-miR-320a |
| hsa-miR-129-5p | hsa-miR-191-5p | hsa-miR-299-3p | hsa-miR-373-3p | hsa-miR-520d-3p | hsa-miR-320b |
| hsa-miR-1297 | hsa-miR-192-5p | hsa-miR-29a-3p | hsa-miR-374a-5p | hsa-miR-520e | hsa-miR-320c |
| hsa-miR-130a-3p | hsa-miR-193a-3p | hsa-miR-29b-3p | hsa-miR-374b-5p | hsa-miR-542-3p | hsa-miR-410-3p |
| hsa-miR-130b-3p | hsa-miR-193b-3p | hsa-miR-29c-3p | hsa-miR-375 | hsa-miR-543 | hsa-miR-411-5p |
| hsa-miR-132-3p | hsa-miR-194-5p | hsa-miR-300 | hsa-miR-376a-3p | hsa-miR-590-5p | hsa-miR-421 |
| hsa-miR-133a-3p | hsa-miR-195-5p | hsa-miR-301a-3p | hsa-miR-376b-3p | hsa-miR-613 | hsa-miR-422a |
| hsa-miR-133b | hsa-miR-196a-5p | hsa-miR-301b-3p | hsa-miR-376c-3p | hsa-miR-615-3p | hsa-miR-424-5p |
| hsa-miR-134-5p | hsa-miR-196b-5p | hsa-miR-302a-3p | hsa-miR-377-3p | hsa-miR-7-5p | hsa-miR-425-5p |
| hsa-miR-135a-5p | hsa-miR-199a-5p | hsa-miR-302b-3p | hsa-miR-378a-3p | hsa-miR-708-5p | hsa-miR-429 |
| hsa-miR-135b-5p | hsa-miR-199b-5p | hsa-miR-302c-3p | hsa-miR-379-5p | hsa-miR-758-3p | hsa-miR-431-5p |
| hsa-miR-136-5p | hsa-miR-19a-3p | hsa-miR-302d-3p | hsa-miR-381-3p | hsa-miR-873-5p | hsa-miR-9-5p |
| hsa-miR-137 | hsa-miR-19b-3p | hsa-miR-302e | hsa-miR-382-5p | hsa-miR-874-3p | hsa-miR-92a-3p |
| hsa-miR-138-5p | hsa-miR-200a-3p | hsa-miR-30a-5p | hsa-miR-383-5p | hsa-miR-875-5p | hsa-miR-92b-3p |
| hsa-miR-139-5p | hsa-miR-200b-3p | hsa-miR-30b-5p | hsa-miR-384 | hsa-miR-876-5p | hsa-miR-93-5p |
| hsa-miR-96-5p | hsa-miR-98-5p | hsa-miR-99a-5p | hsa-miR-99b-5p |  |  |

Table S2. 15 overlapping miRNA target genes in the ceRNA network.

| miRNA target genes | | | | | |
| --- | --- | --- | --- | --- | --- |
| FERMT2 | FGF2 | TGFBR3 | MSI2 | TXNIP | BOLA3 |
| PFN2 | PTX3 | PTGFRN | HOXB2 | SLC16A1 | HMGA2 |
| TMPO | TWF1 | SESN3 | RCAN1 | DBN1 | EIF4G1 |
| F2RL1 | RAPGEF2 | ERO1A | QKI | COL1A1 | TFDP1 |
| SOBP | LRIG3 | NCAPD3 | GDAP1 | LYPLA1 | KIF14 |
| CLDN12 | ADRB1 | TRPS1 | HOXA10 | CA12 | SPAST |
| LCOR | POLR1C | SNX30 | FAM241A | FZD3 | MEX3A |
| TPD52 | MEX3B | CAMK2N1 | IL6ST | WNT5A | PREX1 |
| MARS2 | TTYH3 | HMGA1 | CSTF2 | GCLC | CCNE1 |
| PRKCE | CTSS | LRRC4B | SLC39A14 | EFNA3 | FXR1 |
| CFL2 | CLNS1A | IGF2BP2 | ETS1 | PDCD10 | CRIM1 |
| JAG2 | XPOT | MMS22L | E2F3 | NFIX | S1PR1 |
| VAMP2 | TMEM41A | ZIC5 | GATA2 | TACC1 | SLC12A5 |
| KCTD12 | CBX3 | HOXB7 | BMPR2 | SEMA4C | FSCN1 |
| CALU | TGFBR2 | DPYSL2 | CRY2 | TGFA | EFEMP1 |
| KLF13 | PLPP3 | TET3 | BASP1 | MASTL | MTCL1 |
| BTG2 | TBL1XR1 | MAP3K3 | ADGRL2 | PARD6B | FOXM1 |
| FAM117B | IGF2BP3 | RPS10-NUDT3 | HSP90B1 | HOXD13 | ZCCHC24 |
| PPM1H | SIAH2 | PALD1 | NDC1 | ANLN | XPO5 |
| TCF3 | PRPS2 | TSPAN12 | EIF4EBP1 | SUV39H1 | ADORA2B |
| RTKN2 | MDK | HOXA1 | NSMF | KIF20A | ZNF280C |
| SORT1 | JCAD | NCKAP5 | MAPK6 | RCC2 | SULF2 |
| COL3A1 | CTPS1 | SOX4 | RASSF8 | AK4 | EZH2 |
| XPO1 | PSMD11 | BCL11B | COL1A2 | NME4 | HOXC8 |
| B4GALT3 | KIF26A | HELLS | JAG1 | PLP2 | GAN |
| SALL1 | NOP2 | SMAD7 | ADM | NLN | GATA6 |
| U2SURP | CLGN | NR4A2 | HILPDA | TMEM267 | KLF6 |
| MAP1B | RAD9A | PTPRG | PABPC1 | SSX2IP | NR4A1 |
| GCLM | NCBP2 | SASH1 | LOXL2 | STMN1 | FOXD1 |
| VMP1 | PDK1 | SRPK1 | SH2B3 | CKAP5 | KIF23 |
| ZNF146 | ANKRD13B | KIAA1549 | HEG1 | MNX1 | YWHAZ |
| STARD13 | SLC16A9 | GAB1 | ATAD2 | SMAD6 | BMP2 |
| GNPNAT1 | PRDM5 | TSC22D3 | RAPGEFL1 | HOXA5 | ATP1B1 |
| PRKAA2 | DLX1 | DCDC2 | CDK5R1 | SLC2A1 | AFF3 |
| WWC2 | MAGI3 | GPR37 | HOOK1 | EN2 | EMP2 |
| IGDCC4 | TNC | VIM | CCL2 | HOXA9 | NRARP |
| RNF144B | AMMECR1 | SLC6A8 | SYT14 | STK39 | MAP2K6 |
| PITX2 | MME | CPD | DENR | METAP1 | MEX3D |
| PCDHA12 | FOXQ1 | CCNE2 | DLC1 | ABCC1 | CENPK |
| HSPB8 | PDIA6 | C15orf41 | FUT8 |  |  |

Table S3. The biological functions of differently expressed genes of ceRNA network via the GO annotation.

| Ontology | ID | Description | P value | Count |
| --- | --- | --- | --- | --- |
| BP | GO:0001667 | ameboidal-type cell migration | 5.42E-08 | 22 |
| BP | GO:0060485 | mesenchyme development | 5.88E-08 | 17 |
| BP | GO:0010631 | epithelial cell migration | 6.59E-08 | 19 |
| BP | GO:0090132 | epithelium migration | 7.53E-08 | 19 |
| BP | GO:0070482 | response to oxygen levels | 8.03E-08 | 20 |
| BP | GO:0001666 | response to hypoxia | 8.98E-08 | 19 |
| BP | GO:0090130 | tissue migration | 9.79E-08 | 19 |
| BP | GO:0003279 | cardiac septum development | 1.20E-07 | 11 |
| BP | GO:0036293 | response to decreased oxygen levels | 1.44E-07 | 19 |
| BP | GO:0030900 | forebrain development | 2.26E-07 | 19 |
| BP | GO:1901342 | regulation of vasculature development | 2.43E-07 | 20 |
| BP | GO:0045765 | regulation of angiogenesis | 2.45E-07 | 19 |
| BP | GO:0003281 | ventricular septum development | 3.10E-07 | 9 |
| BP | GO:0060411 | cardiac septum morphogenesis | 3.48E-07 | 9 |
| BP | GO:0061138 | morphogenesis of a branching epithelium | 3.67E-07 | 13 |
| BP | GO:0035270 | endocrine system development | 4.35E-07 | 11 |
| BP | GO:0060562 | epithelial tube morphogenesis | 4.53E-07 | 17 |
| BP | GO:0010632 | regulation of epithelial cell migration | 5.83E-07 | 16 |
| BP | GO:0090068 | positive regulation of cell cycle process | 7.99E-07 | 16 |
| BP | GO:0001763 | morphogenesis of a branching structure | 8.54E-07 | 13 |
| BP | GO:1904018 | positive regulation of vasculature development | 9.05E-07 | 14 |
| BP | GO:0001837 | epithelial to mesenchymal transition | 1.24E-06 | 11 |
| BP | GO:0061448 | connective tissue development | 1.34E-06 | 15 |
| BP | GO:0045766 | positive regulation of angiogenesis | 1.34E-06 | 13 |
| BP | GO:0003002 | regionalization | 1.49E-06 | 17 |
| BP | GO:0048732 | gland development | 1.61E-06 | 19 |
| BP | GO:0051216 | cartilage development | 1.76E-06 | 13 |
| BP | GO:0048754 | branching morphogenesis of an epithelial tube | 2.28E-06 | 11 |
| BP | GO:0010718 | positive regulation of epithelial to mesenchymal transition | 2.29E-06 | 7 |
| BP | GO:0009952 | anterior/posterior pattern specification | 2.95E-06 | 13 |
| BP | GO:0001655 | urogenital system development | 3.02E-06 | 16 |
| BP | GO:0048762 | mesenchymal cell differentiation | 3.10E-06 | 13 |
| BP | GO:0048706 | embryonic skeletal system development | 3.26E-06 | 10 |
| BP | GO:0003007 | heart morphogenesis | 3.66E-06 | 14 |
| BP | GO:0001935 | endothelial cell proliferation | 3.99E-06 | 12 |
| BP | GO:0003206 | cardiac chamber morphogenesis | 4.03E-06 | 10 |
| BP | GO:0003231 | cardiac ventricle development | 4.32E-06 | 10 |
| BP | GO:0003151 | outflow tract morphogenesis | 5.24E-06 | 8 |
| BP | GO:0045787 | positive regulation of cell cycle | 5.92E-06 | 17 |
| BP | GO:0043542 | endothelial cell migration | 7.00E-06 | 14 |
| BP | GO:0003205 | cardiac chamber development | 8.09E-06 | 11 |
| BP | GO:0010634 | positive regulation of epithelial cell migration | 8.09E-06 | 11 |
| BP | GO:0003181 | atrioventricular valve morphogenesis | 8.95E-06 | 5 |
| BP | GO:0001938 | positive regulation of endothelial cell proliferation | 9.20E-06 | 9 |
| BP | GO:0007389 | pattern specification process | 9.36E-06 | 18 |
| BP | GO:0043523 | regulation of neuron apoptotic process | 1.05E-05 | 12 |
| BP | GO:0001936 | regulation of endothelial cell proliferation | 1.06E-05 | 11 |
| BP | GO:0021983 | pituitary gland development | 1.10E-05 | 6 |
| BP | GO:0021537 | telencephalon development | 1.19E-05 | 13 |
| BP | GO:0043524 | negative regulation of neuron apoptotic process | 1.29E-05 | 10 |
| BP | GO:0003171 | atrioventricular valve development | 1.36E-05 | 5 |
| BP | GO:0010717 | regulation of epithelial to mesenchymal transition | 1.39E-05 | 8 |
| BP | GO:0060412 | ventricular septum morphogenesis | 1.45E-05 | 6 |
| BP | GO:0071774 | response to fibroblast growth factor | 1.54E-05 | 10 |
| BP | GO:0050678 | regulation of epithelial cell proliferation | 1.66E-05 | 16 |
| BP | GO:0071230 | cellular response to amino acid stimulus | 1.85E-05 | 7 |
| BP | GO:0045444 | fat cell differentiation | 1.92E-05 | 12 |
| BP | GO:0002062 | chondrocyte differentiation | 1.96E-05 | 9 |
| BP | GO:0034698 | response to gonadotropin | 1.99E-05 | 5 |
| BP | GO:0050769 | positive regulation of neurogenesis | 2.12E-05 | 18 |
| BP | GO:0001764 | neuron migration | 2.28E-05 | 10 |
| BP | GO:0001657 | ureteric bud development | 2.41E-05 | 8 |
| BP | GO:0050673 | epithelial cell proliferation | 2.44E-05 | 17 |
| BP | GO:0010594 | regulation of endothelial cell migration | 2.49E-05 | 12 |
| BP | GO:0072163 | mesonephric epithelium development | 2.60E-05 | 8 |
| BP | GO:0072164 | mesonephric tubule development | 2.60E-05 | 8 |
| BP | GO:0061035 | regulation of cartilage development | 2.70E-05 | 7 |
| BP | GO:0010595 | positive regulation of endothelial cell migration | 2.70E-05 | 9 |
| BP | GO:0021879 | forebrain neuron differentiation | 2.73E-05 | 6 |
| BP | GO:0060840 | artery development | 3.01E-05 | 8 |
| BP | GO:1901214 | regulation of neuron death | 3.10E-05 | 14 |
| BP | GO:0060560 | developmental growth involved in morphogenesis | 3.21E-05 | 12 |
| BP | GO:0061013 | regulation of mRNA catabolic process | 3.34E-05 | 11 |
| BP | GO:0042476 | odontogenesis | 3.44E-05 | 9 |
| BP | GO:0001823 | mesonephros development | 3.47E-05 | 8 |
| BP | GO:0021536 | diencephalon development | 3.52E-05 | 7 |
| BP | GO:0051402 | neuron apoptotic process | 3.79E-05 | 12 |
| BP | GO:0048844 | artery morphogenesis | 3.84E-05 | 7 |
| BP | GO:0003179 | heart valve morphogenesis | 3.86E-05 | 6 |
| BP | GO:0031667 | response to nutrient levels | 4.15E-05 | 18 |
| BP | GO:0061036 | positive regulation of cartilage development | 4.57E-05 | 5 |
| BP | GO:0001570 | vasculogenesis | 4.94E-05 | 7 |
| BP | GO:1901215 | negative regulation of neuron death | 5.00E-05 | 11 |
| BP | GO:0032535 | regulation of cellular component size | 5.02E-05 | 15 |
| BP | GO:0071229 | cellular response to acid chemical | 5.22E-05 | 11 |
| BP | GO:0009612 | response to mechanical stimulus | 5.46E-05 | 11 |
| BP | GO:0072073 | kidney epithelium development | 5.47E-05 | 9 |
| BP | GO:0051261 | protein depolymerization | 5.59E-05 | 8 |
| BP | GO:0071560 | cellular response to transforming growth factor beta stimulus | 5.64E-05 | 12 |
| BP | GO:0071371 | cellular response to gonadotropin stimulus | 5.70E-05 | 4 |
| BP | GO:0071902 | positive regulation of protein serine/threonine kinase activity | 6.27E-05 | 14 |
| BP | GO:0060415 | muscle tissue morphogenesis | 6.28E-05 | 7 |
| BP | GO:0043488 | regulation of mRNA stability | 6.34E-05 | 10 |
| BP | GO:0044344 | cellular response to fibroblast growth factor stimulus | 6.45E-05 | 9 |
| BP | GO:0072001 | renal system development | 6.48E-05 | 13 |
| BP | GO:2000027 | regulation of animal organ morphogenesis | 6.57E-05 | 12 |
| BP | GO:0071559 | response to transforming growth factor beta | 7.08E-05 | 12 |
| BP | GO:0021872 | forebrain generation of neurons | 7.96E-05 | 6 |
| BP | GO:0021953 | central nervous system neuron differentiation | 8.38E-05 | 10 |
| BP | GO:0043487 | regulation of RNA stability | 8.38E-05 | 10 |
| BP | GO:0051781 | positive regulation of cell division | 9.17E-05 | 7 |
| BP | GO:0048638 | regulation of developmental growth | 9.42E-05 | 14 |
| BP | GO:0003170 | heart valve development | 9.61E-05 | 6 |
| BP | GO:0070997 | neuron death | 9.71E-05 | 14 |
| BP | GO:0048644 | muscle organ morphogenesis | 9.86E-05 | 7 |
| BP | GO:0007178 | transmembrane receptor protein serine/threonine kinase signaling pathway | 0.000100052 | 14 |
| BP | GO:0030902 | hindbrain development | 0.000103231 | 9 |
| BP | GO:0031668 | cellular response to extracellular stimulus | 0.000113524 | 12 |
| BP | GO:0006403 | RNA localization | 0.000122952 | 11 |
| BP | GO:0007219 | Notch signaling pathway | 0.000130185 | 10 |
| BP | GO:0050657 | nucleic acid transport | 0.000130185 | 10 |
| BP | GO:0050658 | RNA transport | 0.000130185 | 10 |
| BP | GO:0060389 | pathway-restricted SMAD protein phosphorylation | 0.000137286 | 6 |
| BP | GO:0051236 | establishment of RNA localization | 0.000147722 | 10 |
| BP | GO:0060993 | kidney morphogenesis | 0.000149499 | 7 |
| BP | GO:0007517 | muscle organ development | 0.000156942 | 15 |
| BP | GO:0001822 | kidney development | 0.000159911 | 12 |
| BP | GO:0060541 | respiratory system development | 0.000160488 | 10 |
| BP | GO:0045746 | negative regulation of Notch signaling pathway | 0.000167956 | 5 |
| BP | GO:0030516 | regulation of axon extension | 0.000170553 | 7 |
| BP | GO:1903311 | regulation of mRNA metabolic process | 0.000176659 | 13 |
| BP | GO:0062009 | secondary palate development | 0.000186798 | 4 |
| BP | GO:0007019 | microtubule depolymerization | 0.000187628 | 5 |
| BP | GO:0045666 | positive regulation of neuron differentiation | 0.000189171 | 14 |
| BP | GO:0055008 | cardiac muscle tissue morphogenesis | 0.000191366 | 6 |
| BP | GO:0002042 | cell migration involved in sprouting angiogenesis | 0.000193944 | 7 |
| BP | GO:0003183 | mitral valve morphogenesis | 0.000199847 | 3 |
| BP | GO:0021877 | forebrain neuron fate commitment | 0.000199847 | 3 |
| BP | GO:0070341 | fat cell proliferation | 0.000199847 | 3 |
| BP | GO:0070344 | regulation of fat cell proliferation | 0.000199847 | 3 |
| BP | GO:0007409 | axonogenesis | 0.000205732 | 16 |
| BP | GO:0051302 | regulation of cell division | 0.000220062 | 9 |
| BP | GO:0050679 | positive regulation of epithelial cell proliferation | 0.000221257 | 10 |
| BP | GO:0048562 | embryonic organ morphogenesis | 0.000221649 | 12 |
| BP | GO:0021543 | pallium development | 0.000230026 | 9 |
| BP | GO:0071772 | response to BMP | 0.000240362 | 9 |
| BP | GO:0071773 | cellular response to BMP stimulus | 0.000240362 | 9 |
| BP | GO:0060070 | canonical Wnt signaling pathway | 0.000244531 | 13 |
| BP | GO:0048568 | embryonic organ development | 0.000249399 | 15 |
| BP | GO:0003208 | cardiac ventricle morphogenesis | 0.000261007 | 6 |
| BP | GO:0030324 | lung development | 0.000262192 | 9 |
| BP | GO:0003174 | mitral valve development | 0.000272326 | 3 |
| BP | GO:0071496 | cellular response to external stimulus | 0.000274218 | 13 |
| BP | GO:0008593 | regulation of Notch signaling pathway | 0.000297017 | 7 |
| BP | GO:0003148 | outflow tract septum morphogenesis | 0.000299822 | 4 |
| BP | GO:0001101 | response to acid chemical | 0.000306935 | 13 |
| BP | GO:0032956 | regulation of actin cytoskeleton organization | 0.000306935 | 13 |
| BP | GO:0030323 | respiratory tube development | 0.000310802 | 9 |
| BP | GO:0090596 | sensory organ morphogenesis | 0.00031113 | 11 |
| BP | GO:0018105 | peptidyl-serine phosphorylation | 0.000311979 | 12 |
| BP | GO:0010769 | regulation of cell morphogenesis involved in differentiation | 0.000331374 | 12 |
| BP | GO:0043406 | positive regulation of MAP kinase activity | 0.000332426 | 11 |
| BP | GO:0072006 | nephron development | 0.000350585 | 8 |
| BP | GO:0008361 | regulation of cell size | 0.00035196 | 9 |
| BP | GO:0030177 | positive regulation of Wnt signaling pathway | 0.00035196 | 9 |
| BP | GO:0048736 | appendage development | 0.00035196 | 9 |
| BP | GO:0060173 | limb development | 0.00035196 | 9 |
| BP | GO:0071156 | regulation of cell cycle arrest | 0.000352868 | 7 |
| BP | GO:0043583 | ear development | 0.000360716 | 10 |
| BP | GO:0043534 | blood vessel endothelial cell migration | 0.00036664 | 9 |
| BP | GO:0021761 | limbic system development | 0.000373248 | 7 |
| BP | GO:0072009 | nephron epithelium development | 0.000373248 | 7 |
| BP | GO:0061387 | regulation of extent of cell growth | 0.00039456 | 7 |
| BP | GO:1902903 | regulation of supramolecular fiber organization | 0.000403641 | 13 |
| BP | GO:0002040 | sprouting angiogenesis | 0.000413743 | 9 |
| BP | GO:1904019 | epithelial cell apoptotic process | 0.000416834 | 7 |
| BP | GO:0010975 | regulation of neuron projection development | 0.000418808 | 16 |
| BP | GO:0002065 | columnar/cuboidal epithelial cell differentiation | 0.000440102 | 7 |
| BP | GO:0060419 | heart growth | 0.000440102 | 7 |
| BP | GO:0003209 | cardiac atrium morphogenesis | 0.000455004 | 4 |
| BP | GO:0021766 | hippocampus development | 0.000459093 | 6 |
| BP | GO:0110110 | positive regulation of animal organ morphogenesis | 0.000459093 | 6 |
| BP | GO:0043200 | response to amino acid | 0.000464395 | 7 |
| BP | GO:0071158 | positive regulation of cell cycle arrest | 0.000490379 | 6 |
| BP | GO:0090049 | regulation of cell migration involved in sprouting angiogenesis | 0.000490379 | 6 |
| BP | GO:0031098 | stress-activated protein kinase signaling cascade | 0.000497943 | 12 |
| BP | GO:0045165 | cell fate commitment | 0.000502966 | 11 |
| BP | GO:0034694 | response to prostaglandin | 0.000517467 | 4 |
| BP | GO:0030111 | regulation of Wnt signaling pathway | 0.000525135 | 13 |
| BP | GO:0009743 | response to carbohydrate | 0.000529982 | 10 |
| BP | GO:2001212 | regulation of vasculogenesis | 0.000584775 | 3 |
| BP | GO:0060317 | cardiac epithelial to mesenchymal transition | 0.000585795 | 4 |
| BP | GO:0048839 | inner ear development | 0.000585835 | 9 |
| BP | GO:0016358 | dendrite development | 0.000586141 | 10 |
| BP | GO:0042471 | ear morphogenesis | 0.000602404 | 7 |
| BP | GO:0018209 | peptidyl-serine modification | 0.000604735 | 12 |
| BP | GO:0030509 | BMP signaling pathway | 0.000683657 | 8 |
| BP | GO:0048675 | axon extension | 0.000699683 | 7 |
| BP | GO:0034616 | response to laminar fluid shear stress | 0.000724432 | 3 |
| BP | GO:0031099 | regeneration | 0.000730224 | 9 |
| BP | GO:0098727 | maintenance of cell number | 0.000742778 | 8 |
| BP | GO:0001658 | branching involved in ureteric bud morphogenesis | 0.000746215 | 5 |
| BP | GO:0097755 | positive regulation of blood vessel diameter | 0.000746215 | 5 |
| BP | GO:0007179 | transforming growth factor beta receptor signaling pathway | 0.000756893 | 9 |
| BP | GO:0015931 | nucleobase-containing compound transport | 0.000760417 | 10 |
| BP | GO:0042475 | odontogenesis of dentin-containing tooth | 0.000804573 | 6 |
| BP | GO:0032147 | activation of protein kinase activity | 0.00081112 | 12 |
| BP | GO:0003230 | cardiac atrium development | 0.000828988 | 4 |
| BP | GO:0030510 | regulation of BMP signaling pathway | 0.000852761 | 6 |
| BP | GO:0051271 | negative regulation of cellular component movement | 0.000883432 | 13 |
| BP | GO:0032354 | response to follicle-stimulating hormone | 0.000883639 | 3 |
| BP | GO:0097067 | cellular response to thyroid hormone stimulus | 0.000883639 | 3 |
| BP | GO:2001224 | positive regulation of neuron migration | 0.000883639 | 3 |
| BP | GO:0022604 | regulation of cell morphogenesis | 0.000893086 | 15 |
| BP | GO:0043405 | regulation of MAP kinase activity | 0.000899465 | 12 |
| BP | GO:0000578 | embryonic axis specification | 0.000923801 | 4 |
| BP | GO:0090287 | regulation of cellular response to growth factor stimulus | 0.000929999 | 11 |
| BP | GO:0043434 | response to peptide hormone | 0.000941739 | 14 |
| BP | GO:0003073 | regulation of systemic arterial blood pressure | 0.00095579 | 6 |
| BP | GO:0072080 | nephron tubule development | 0.00095579 | 6 |
| BP | GO:0032970 | regulation of actin filament-based process | 0.0009709 | 13 |
| BP | GO:0071456 | cellular response to hypoxia | 0.001000144 | 9 |
| BP | GO:0048511 | rhythmic process | 0.001010293 | 11 |
| BP | GO:0090050 | positive regulation of cell migration involved in sprouting angiogenesis | 0.001025995 | 4 |
| BP | GO:0061326 | renal tubule development | 0.001068136 | 6 |
| BP | GO:0048663 | neuron fate commitment | 0.00116075 | 5 |
| BP | GO:0060675 | ureteric bud morphogenesis | 0.00116075 | 5 |
| BP | GO:0003158 | endothelium development | 0.001167402 | 7 |
| BP | GO:0072171 | mesonephric tubule morphogenesis | 0.00124364 | 5 |
| BP | GO:0032506 | cytokinetic process | 0.001253747 | 4 |
| BP | GO:2000826 | regulation of heart morphogenesis | 0.001253747 | 4 |
| BP | GO:0042472 | inner ear morphogenesis | 0.001255334 | 6 |
| BP | GO:0048565 | digestive tract development | 0.001273962 | 7 |
| BP | GO:0007015 | actin filament organization | 0.001278017 | 13 |
| BP | GO:0060326 | cell chemotaxis | 0.001286274 | 11 |
| BP | GO:0006979 | response to oxidative stress | 0.001298283 | 14 |
| BP | GO:0001889 | liver development | 0.001330021 | 7 |
| BP | GO:0050921 | positive regulation of chemotaxis | 0.001330021 | 7 |
| BP | GO:0031589 | cell-substrate adhesion | 0.001369618 | 12 |
| BP | GO:0032467 | positive regulation of cytokinesis | 0.001379912 | 4 |
| BP | GO:0110053 | regulation of actin filament organization | 0.001389042 | 10 |
| BP | GO:0036294 | cellular response to decreased oxygen levels | 0.001389626 | 9 |
| BP | GO:0050920 | regulation of chemotaxis | 0.001389626 | 9 |
| BP | GO:0007584 | response to nutrient | 0.001480505 | 9 |
| BP | GO:0035728 | response to hepatocyte growth factor | 0.001488423 | 3 |
| BP | GO:0061008 | hepaticobiliary system development | 0.001509842 | 7 |
| BP | GO:0060537 | muscle tissue development | 0.00152475 | 13 |
| BP | GO:0034329 | cell junction assembly | 0.001558201 | 13 |
| BP | GO:2001222 | regulation of neuron migration | 0.001658335 | 4 |
| BP | GO:0006402 | mRNA catabolic process | 0.001730536 | 12 |
| BP | GO:0060039 | pericardium development | 0.001735471 | 3 |
| BP | GO:0060216 | definitive hemopoiesis | 0.001735471 | 3 |
| BP | GO:0060602 | branch elongation of an epithelium | 0.001735471 | 3 |
| BP | GO:0061162 | establishment of monopolar cell polarity | 0.001735471 | 3 |
| BP | GO:1901881 | positive regulation of protein depolymerization | 0.001735471 | 3 |
| BP | GO:0008217 | regulation of blood pressure | 0.00176719 | 8 |
| BP | GO:0001841 | neural tube formation | 0.001789311 | 6 |
| BP | GO:0055017 | cardiac muscle tissue growth | 0.001789311 | 6 |
| BP | GO:0000281 | mitotic cytokinesis | 0.001835895 | 5 |
| BP | GO:0035296 | regulation of tube diameter | 0.001851093 | 7 |
| BP | GO:0097746 | regulation of blood vessel diameter | 0.001851093 | 7 |
| BP | GO:0048639 | positive regulation of developmental growth | 0.001892632 | 8 |
| BP | GO:2000045 | regulation of G1/S transition of mitotic cell cycle | 0.001892632 | 8 |
| BP | GO:0030198 | extracellular matrix organization | 0.001895277 | 12 |
| BP | GO:0035150 | regulation of tube size | 0.001925995 | 7 |
| BP | GO:0043062 | extracellular structure organization | 0.001938415 | 12 |
| BP | GO:0031100 | animal organ regeneration | 0.001951695 | 5 |
| BP | GO:0003197 | endocardial cushion development | 0.001973551 | 4 |
| BP | GO:0046189 | phenol-containing compound biosynthetic process | 0.001973551 | 4 |
| BP | GO:0007568 | aging | 0.001976564 | 11 |
| BP | GO:0032386 | regulation of intracellular transport | 0.001982355 | 12 |
| BP | GO:0003177 | pulmonary valve development | 0.002006671 | 3 |
| BP | GO:0035162 | embryonic hemopoiesis | 0.002006671 | 3 |
| BP | GO:0061339 | establishment or maintenance of monopolar cell polarity | 0.002006671 | 3 |
| BP | GO:0032388 | positive regulation of intracellular transport | 0.002009302 | 9 |
| BP | GO:0072078 | nephron tubule morphogenesis | 0.002072747 | 5 |
| BP | GO:0055123 | digestive system development | 0.002082832 | 7 |
| BP | GO:0007411 | axon guidance | 0.002096367 | 10 |
| BP | GO:0006417 | regulation of translation | 0.002137691 | 13 |
| BP | GO:0034248 | regulation of cellular amide metabolic process | 0.002141353 | 14 |
| BP | GO:0031018 | endocrine pancreas development | 0.002145701 | 4 |
| BP | GO:0048483 | autonomic nervous system development | 0.002145701 | 4 |
| BP | GO:0097485 | neuron projection guidance | 0.002152282 | 10 |
| BP | GO:0035107 | appendage morphogenesis | 0.002249373 | 7 |
| BP | GO:0035108 | limb morphogenesis | 0.002249373 | 7 |
| BP | GO:0035148 | tube formation | 0.002249373 | 7 |
| BP | GO:0048738 | cardiac muscle tissue development | 0.002258973 | 9 |
| BP | GO:0000082 | G1/S transition of mitotic cell cycle | 0.002267717 | 10 |
| BP | GO:0003094 | glomerular filtration | 0.002302819 | 3 |
| BP | GO:0035024 | negative regulation of Rho protein signal transduction | 0.002302819 | 3 |
| BP | GO:0060065 | uterus development | 0.002302819 | 3 |
| BP | GO:0071379 | cellular response to prostaglandin stimulus | 0.002302819 | 3 |
| BP | GO:0071453 | cellular response to oxygen levels | 0.002325095 | 9 |
| BP | GO:0002067 | glandular epithelial cell differentiation | 0.002327939 | 4 |
| BP | GO:0072088 | nephron epithelium morphogenesis | 0.002331157 | 5 |
| BP | GO:0050773 | regulation of dendrite development | 0.002336406 | 7 |
| BP | GO:0043254 | regulation of protein-containing complex assembly | 0.002366403 | 13 |
| BP | GO:0016331 | morphogenesis of embryonic epithelium | 0.002426013 | 7 |
| BP | GO:0061351 | neural precursor cell proliferation | 0.002426013 | 7 |
| BP | GO:0048608 | reproductive structure development | 0.002463349 | 13 |
| BP | GO:0031109 | microtubule polymerization or depolymerization | 0.002480094 | 6 |
| BP | GO:0045646 | regulation of erythrocyte differentiation | 0.002520558 | 4 |
| BP | GO:0061383 | trabecula morphogenesis | 0.002520558 | 4 |
| BP | GO:0061614 | pri-miRNA transcription by RNA polymerase II | 0.002520558 | 4 |
| BP | GO:0040013 | negative regulation of locomotion | 0.002521978 | 12 |
| BP | GO:0007050 | cell cycle arrest | 0.002532748 | 9 |
| BP | GO:0031669 | cellular response to nutrient levels | 0.002532748 | 9 |
| BP | GO:0032872 | regulation of stress-activated MAPK cascade | 0.002532748 | 9 |
| BP | GO:0035690 | cellular response to drug | 0.002563535 | 13 |
| BP | GO:0021700 | developmental maturation | 0.002578215 | 10 |
| BP | GO:0021987 | cerebral cortex development | 0.002593123 | 6 |
| BP | GO:0046620 | regulation of organ growth | 0.002593123 | 6 |
| BP | GO:0048640 | negative regulation of developmental growth | 0.002593123 | 6 |
| BP | GO:0046578 | regulation of Ras protein signal transduction | 0.002605145 | 9 |
| BP | GO:0021954 | central nervous system neuron development | 0.002612241 | 5 |
| BP | GO:0061333 | renal tubule morphogenesis | 0.002612241 | 5 |
| BP | GO:0072028 | nephron morphogenesis | 0.002612241 | 5 |
| BP | GO:0000187 | activation of MAPK activity | 0.002613152 | 7 |
| BP | GO:0061458 | reproductive system development | 0.002614868 | 13 |
| BP | GO:0034695 | response to prostaglandin E | 0.002624668 | 3 |
| BP | GO:0097205 | renal filtration | 0.002624668 | 3 |
| BP | GO:0031346 | positive regulation of cell projection organization | 0.00263189 | 12 |
| BP | GO:0070302 | regulation of stress-activated protein kinase signaling cascade | 0.002679174 | 9 |
| BP | GO:0030218 | erythrocyte differentiation | 0.002709978 | 6 |
| BP | GO:0045446 | endothelial cell differentiation | 0.002709978 | 6 |
| BP | GO:0030168 | platelet activation | 0.002710786 | 7 |
| BP | GO:0051403 | stress-activated MAPK cascade | 0.002711578 | 10 |
| BP | GO:0060828 | regulation of canonical Wnt signaling pathway | 0.002711578 | 10 |
| BP | GO:0043536 | positive regulation of blood vessel endothelial cell migration | 0.002761636 | 5 |
| BP | GO:0090092 | regulation of transmembrane receptor protein serine/threonine kinase signaling pathway | 0.002832231 | 9 |
| BP | GO:0009749 | response to glucose | 0.002888788 | 8 |
| BP | GO:1903707 | negative regulation of hemopoiesis | 0.002914438 | 7 |
| BP | GO:0007162 | negative regulation of cell adhesion | 0.00292193 | 10 |
| BP | GO:0034629 | cellular protein-containing complex localization | 0.002972935 | 3 |
| BP | GO:0043302 | positive regulation of leukocyte degranulation | 0.002972935 | 3 |
| BP | GO:0060148 | positive regulation of posttranscriptional gene silencing | 0.002972935 | 3 |
| BP | GO:2000178 | negative regulation of neural precursor cell proliferation | 0.002972935 | 3 |
| BP | GO:0043535 | regulation of blood vessel endothelial cell migration | 0.00302056 | 7 |
| BP | GO:0014706 | striated muscle tissue development | 0.003047738 | 12 |
| BP | GO:0019827 | stem cell population maintenance | 0.003129616 | 7 |
| BP | GO:1901990 | regulation of mitotic cell cycle phase transition | 0.003176121 | 13 |
| BP | GO:0009746 | response to hexose | 0.003365726 | 8 |
| BP | GO:1902806 | regulation of cell cycle G1/S phase transition | 0.003365726 | 8 |
| BP | GO:0045599 | negative regulation of fat cell differentiation | 0.003400574 | 4 |
| BP | GO:0090102 | cochlea development | 0.003400574 | 4 |
| BP | GO:1901879 | regulation of protein depolymerization | 0.003421504 | 5 |
| BP | GO:1903845 | negative regulation of cellular response to transforming growth factor beta stimulus | 0.003421504 | 5 |
| BP | GO:0030278 | regulation of ossification | 0.003468047 | 8 |
| BP | GO:0048285 | organelle fission | 0.00349155 | 13 |
| BP | GO:0022612 | gland morphogenesis | 0.003495936 | 6 |
| BP | GO:0006401 | RNA catabolic process | 0.003515402 | 12 |
| BP | GO:0035265 | organ growth | 0.003572767 | 8 |
| BP | GO:0001503 | ossification | 0.003586705 | 12 |
| BP | GO:0060420 | regulation of heart growth | 0.003602743 | 5 |
| BP | GO:0070828 | heterochromatin organization | 0.003602743 | 5 |
| BP | GO:0044843 | cell cycle G1/S phase transition | 0.003632272 | 10 |
| BP | GO:0030514 | negative regulation of BMP signaling pathway | 0.003649373 | 4 |
| BP | GO:0032330 | regulation of chondrocyte differentiation | 0.003649373 | 4 |
| BP | GO:0060421 | positive regulation of heart growth | 0.003649373 | 4 |
| BP | GO:0045992 | negative regulation of embryonic development | 0.003751401 | 3 |
| BP | GO:0051497 | negative regulation of stress fiber assembly | 0.003751401 | 3 |
| BP | GO:0060740 | prostate gland epithelium morphogenesis | 0.003751401 | 3 |
| BP | GO:0097066 | response to thyroid hormone | 0.003751401 | 3 |
| BP | GO:0009798 | axis specification | 0.003790772 | 5 |
| BP | GO:0034101 | erythrocyte homeostasis | 0.003792194 | 6 |
| BP | GO:0097549 | chromatin organization involved in negative regulation of transcription | 0.003792194 | 6 |
| BP | GO:0062197 | cellular response to chemical stress | 0.003830205 | 11 |
| BP | GO:0022407 | regulation of cell-cell adhesion | 0.003883691 | 12 |
| BP | GO:0002064 | epithelial cell development | 0.003901684 | 8 |
| BP | GO:0034284 | response to monosaccharide | 0.003901684 | 8 |
| BP | GO:0002043 | blood vessel endothelial cell proliferation involved in sprouting angiogenesis | 0.003910243 | 4 |
| BP | GO:0060563 | neuroepithelial cell differentiation | 0.003910243 | 4 |
| BP | GO:0030098 | lymphocyte differentiation | 0.00408255 | 11 |
| BP | GO:0001838 | embryonic epithelial tube formation | 0.004106683 | 6 |
| BP | GO:1901623 | regulation of lymphocyte chemotaxis | 0.004182849 | 3 |
| BP | GO:0030199 | collagen fibril organization | 0.004183452 | 4 |
| BP | GO:0090288 | negative regulation of cellular response to growth factor stimulus | 0.004251859 | 7 |
| BP | GO:0030326 | embryonic limb morphogenesis | 0.004270971 | 6 |
| BP | GO:0035113 | embryonic appendage morphogenesis | 0.004270971 | 6 |
| BP | GO:0000280 | nuclear division | 0.004282462 | 12 |
| BP | GO:0048863 | stem cell differentiation | 0.004320963 | 9 |
| BP | GO:2000177 | regulation of neural precursor cell proliferation | 0.004396985 | 5 |
| BP | GO:0018107 | peptidyl-threonine phosphorylation | 0.004440065 | 6 |
| BP | GO:0090101 | negative regulation of transmembrane receptor protein serine/threonine kinase signaling pathway | 0.004440065 | 6 |
| BP | GO:0002011 | morphogenesis of an epithelial sheet | 0.004469267 | 4 |
| BP | GO:1990138 | neuron projection extension | 0.004538015 | 7 |
| BP | GO:0032465 | regulation of cytokinesis | 0.00461356 | 5 |
| BP | GO:0060512 | prostate gland morphogenesis | 0.004643213 | 3 |
| BP | GO:0090344 | negative regulation of cell aging | 0.004643213 | 3 |
| BP | GO:0060688 | regulation of morphogenesis of a branching structure | 0.00476795 | 4 |
| BP | GO:0001843 | neural tube closure | 0.004837618 | 5 |
| BP | GO:0030901 | midbrain development | 0.004837618 | 5 |
| BP | GO:0045778 | positive regulation of ossification | 0.004837618 | 5 |
| BP | GO:0010810 | regulation of cell-substrate adhesion | 0.004893732 | 8 |
| BP | GO:0000910 | cytokinesis | 0.004994225 | 7 |
| BP | GO:0001578 | microtubule bundle formation | 0.005069296 | 5 |
| BP | GO:0060606 | tube closure | 0.005069296 | 5 |
| BP | GO:0060043 | regulation of cardiac muscle cell proliferation | 0.005079758 | 4 |
| BP | GO:0010575 | positive regulation of vascular endothelial growth factor production | 0.005133033 | 3 |
| BP | GO:0015949 | nucleobase-containing small molecule interconversion | 0.005133033 | 3 |
| BP | GO:0032232 | negative regulation of actin filament bundle assembly | 0.005133033 | 3 |
| BP | GO:0032874 | positive regulation of stress-activated MAPK cascade | 0.005153708 | 7 |
| BP | GO:0043624 | cellular protein complex disassembly | 0.005169538 | 8 |
| BP | GO:0032271 | regulation of protein polymerization | 0.005311826 | 8 |
| BP | GO:0070304 | positive regulation of stress-activated protein kinase signaling cascade | 0.005316991 | 7 |
| BP | GO:0035904 | aorta development | 0.005404946 | 4 |
| CC | GO:0000792 | heterochromatin | 3.04E-05 | 7 |
| CC | GO:0030496 | midbody | 3.85E-05 | 10 |
| CC | GO:0005583 | fibrillar collagen trimer | 0.000244339 | 3 |
| CC | GO:0098643 | banded collagen fibril | 0.000244339 | 3 |
| CC | GO:0005667 | transcription regulator complex | 0.000407303 | 14 |
| CC | GO:0005911 | cell-cell junction | 0.00041718 | 14 |
| CC | GO:0045120 | pronucleus | 0.000650826 | 3 |
| CC | GO:0005874 | microtubule | 0.001309718 | 13 |
| CC | GO:0098644 | complex of collagen trimers | 0.001338926 | 3 |
| CC | GO:0005721 | pericentric heterochromatin | 0.001561664 | 3 |
| MF | GO:0046332 | SMAD binding | 7.28E-07 | 9 |
| MF | GO:0001228 | DNA-binding transcription activator activity, RNA polymerase II-specific | 1.21E-05 | 18 |
| MF | GO:0001216 | DNA-binding transcription activator activity | 1.25E-05 | 18 |
| MF | GO:0019838 | growth factor binding | 6.05E-05 | 9 |
| MF | GO:0008017 | microtubule binding | 0.000298583 | 11 |
| MF | GO:0034713 | type I transforming growth factor beta receptor binding | 0.000302341 | 3 |
| MF | GO:0048407 | platelet-derived growth factor binding | 0.000302341 | 3 |
| MF | GO:0015631 | tubulin binding | 0.000352393 | 13 |
| MF | GO:0005160 | transforming growth factor beta receptor binding | 0.000445326 | 5 |
| MF | GO:0019199 | transmembrane receptor protein kinase activity | 0.000483727 | 6 |
| MF | GO:0030165 | PDZ domain binding | 0.000760661 | 6 |
| MF | GO:0005024 | transforming growth factor beta-activated receptor activity | 0.000803228 | 3 |
| MF | GO:0035259 | glucocorticoid receptor binding | 0.000979435 | 3 |
| MF | GO:0004675 | transmembrane receptor protein serine/threonine kinase activity | 0.001178307 | 3 |
| MF | GO:0046965 | retinoid X receptor binding | 0.001178307 | 3 |
| MF | GO:0031435 | mitogen-activated protein kinase kinase kinase binding | 0.001400892 | 3 |
